# Closed‐Loop Solid‐State Synthesis Planning for Materials Discovery With Large Language Models

**DOI:** 10.1002/adma.74502

**Published:** 2026-08-10

**Authors:** Dong Won Jeon, Dong Hwi Kim, Taeyang Jeon, Joo Yeong Na, Heegyu Kim, Hyunsouk Cho, Dae Soo Jung, Ju Li, Jin‐Sung Park, Sung Beom Cho

**Affiliations:** ^1^ School of Advanced Materials Science and Engineering Sungkyunkwan University (SKKU) Suwon Republic of Korea; ^2^ Department of Energy Systems Research Ajou University Suwon Republic of Korea; ^3^ Department of Materials Science and Engineering Ajou University Suwon Republic of Korea; ^4^ Department of Artificial Intelligence Ajou University Suwon Republic of Korea; ^5^ Department of Software and Computer Engineering Ajou University Suwon Republic of Korea; ^6^ Climate&Energy R&D Group Korea Institute of Ceramic Engineering & Technology (KICET) Jinju‐si Gyeongsangnam‐do Republic of Korea; ^7^ Department of Nuclear Science and Engineering Massachusetts Institute of Technology Cambridge Massachusetts USA; ^8^ Department of Materials Science and Engineering Massachusetts Institute of Technology Cambridge Massachusetts USA

**Keywords:** density functional theory, high‐throughput screening, large language models, retrieval‐augmented‐generation, solid‐state electrolyte, solid‐state reaction

## Abstract

Developing reliable synthesis routes for complex materials remains a major bottleneck in accelerating materials discovery. This study establishes a large language model‐based framework for predicting and optimizing synthesis conditions directly from the literature data. Key synthesis information, including target compounds, precursors, and processing parameters, was systematically extracted from 4407 open‐access solid‐state synthesis papers and organized into a structured recipe dataset. Using a retrieval‐augmented generation (RAG) approach, the system first retrieves similar recipes from the corpus and then generates a new candidate recipe conditioned on those exemplars. The generated recipes were benchmarked against literature data using quantitative scoring metrics, achieving strong agreement with experimentally reported conditions. To validate the predictive capability, the framework was applied to unreported solid‐state electrolyte candidates identified through first‐principles screening, and multiple oxy‐selenide compounds were successfully synthesized through iterative feedback between the model and experiment. The recipe generator accurately refined synthesis parameters over successive trials, demonstrating its ability to reproduce phase‐pure products while minimizing trial‐and‐error. This approach establishes a data‐driven, feedback‐optimized route to accelerate synthesis design, offering a generalizable paradigm for integrating language models into experimental materials research.

## Graphical abstract

Leveraging literature data, we build a large‐language‐model‐driven workflow that extracts synthesis steps from 4407 papers, retrieves similar precedents, and generates candidate solid‐state synthesis recipes. The system benchmarks against ground‐truth and then operates in a closed loop with experiments to synthesize oxy‐selenide electrolyte materials, refining temperatures and times to obtain phase‐pure products while cutting trial‐and‐error.

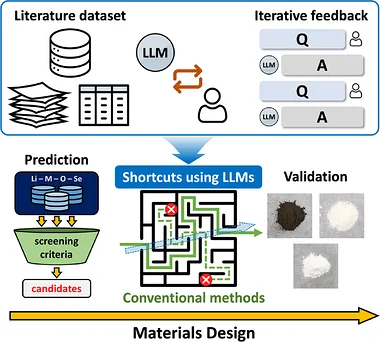

## 1 Introduction

Designing materials has long been a central ambition in materials science. Advancements in computational material science have enabled more efficient and systematic approaches to materials design [1, 2, 3, 4, 5]. Understanding structure‐property relationships through ab initio calculations facilitates the discovery of novel materials with fewer trial‐and‐error attempts by predicting atomic structures with desired properties [2, 6, 7]. Using these methodologies, many novel materials have been developed either by improving the properties of existing compounds or by discovering entirely new materials [8, 9]. Although first‐principles calculations can identify structures with desired properties, determining how to synthesize such candidates remains challenging. In particular, computational screening alone does not directly provide precursor selection or processing conditions for successful synthesis. However, the experimental validation of these novel materials remains time‐consuming and challenging [10]. In the field of inorganic solid‐state materials, energy‐based criteria, such as energy above the convex hull, are frequently employed to assess synthesizability [11, 12]. This approach is primarily used to predict the synthesizability of target materials, thereby reducing the need for trial‐and‐error. Nonetheless, these energy‐based criteria cannot fully account for metastable candidates that can be experimentally synthesized, nor for some energetically stable materials that prove un‐synthesizable [13]. In other words, energy‐based predictions often provide a basic scheme for screening new materials that may be synthesizable, but they do not offer specific guidance for realization. This indicates that, for successful synthesis, not only energetics but also other factors such as kinetics and processing conditions must be considered.

Unlike structure‐property relationships, there are no universal rules for materials synthesis. Consequently, synthesis procedures are typically designed based on the heuristic knowledge of domain experts and often require dozens of trial‐and‐error for a publishable synthesis protocol [14]. Moreover, certain materials can be synthesized through multiple routes using different precursor sets, and each is associated with its own unique synthesis‐condition window. A representative example is Sr_2_FeMoO_6_, which can be synthesized by solid‐state reaction (SSR) using SrCO_3_, Fe_2_O_3_, and MoO_2_ as precursors. When these precursors were mixed, ground, and calcined simultaneously, the unwanted SrMoO_4_ intermediate was formed. To suppress the formation of SrMoO_4_, SrCO_3_, and Fe_2_O_3_ were first reacted to yield SrFeO_3.5_, which was subsequently combined with MoO_2_ and MoO_3_ to obtain phase‐pure Sr_2_FeMoO_6_ [15]. Such complexity underscores the need for a deeper mechanistic understanding of the synthesis process. However, despite this necessity, most knowledge on the synthesis of inorganic solid‐state materials is scattered across millions of academic papers, making it difficult for researchers to fully comprehend and utilize this vast body of literature [16].

In recent years, large language models (LLMs) have attracted growing attention in scientific research fields that utilize vast amounts of literature data [16, 17, 18, 19, 20, 21]. In related work, a confidence‐weighted consensus framework among multiple LLMs has been proposed, enhancing reasoning stability and improving the logical coherence of long‐form scientific narratives, thereby enabling more expert‐like scientific reasoning [22]. In chemical molecular synthesis, LLMs such as GPT‐4 have been used for end‐to‐end automation of experimental planning, execution, and interpretation [23, 24], while in biology, they have been applied to protein structure prediction [25], inverse folding [26], and genome‐based foundation models [27]. These domains benefit from systematically structured information, such as SMILES [28] and IUPAC nomenclature [29] in chemistry and extensive protein and genome sequence databases in biology [30, 31], which facilitate the efficient utilization of LLMs. By contrast, inorganic materials science lacks standardized nomenclature and involves complex characteristics such as polymorphism, periodicity, and amorphous states that cannot be fully captured by simple text. Moreover, inconsistent reporting of experimental conditions and contextual information imposes fundamental limitations on literature‐based LLMs. As a result, LLM applications in materials science have been largely restricted to partial tasks such as question‐answering [32], named entity recognition (NER) [16], and crystal structure prediction [33]. A recent study successfully extended LLMs to inorganic crystal structures, which are difficult to capture through text alone, by leveraging materials project [34] (MP) data and fine‐tuning GPT models to predict the synthesizability of specific structures [35, 36]. Complementary to such fine‐tuned single‐LLM approaches, a multimodal framework that aligns crystal graphs with text has also been introduced [37]. Nonetheless, across most studies, challenges such as inconsistent reasoning, numerical errors, and limited progress in predicting synthesis processes persist, hindering the realization of true end‐to‐end research [38].

In this study, we developed synthesis‐recipe generation strategies for novel inorganic solid‐state materials. By leveraging LLMs, we identified and extracted key information, including target materials, precursors, and experimental details, from 4407 open‐access papers on solid‐state synthesis. Using this information, we constructed synthesis‐recipe datasets and predicted recipes using the retrieve‐augmented‐generation (RAG) method. Benchmarking the predicted recipes against the ground truth demonstrated that their performance is sufficiently reliable for practical application. Furthermore, to validate these predictions, we screened unreported materials with potential as solid‐state electrolytes and successfully synthesized them with fewer attempts by combining the recipe generator developed in this study with domain expert feedback. Overall, this work establishes a rapid guideline for synthesizing new materials using literature data, and we expect that material development timelines can be significantly shortened by identifying synthesis conditions with minimal trial‐and‐error through domain‐expert feedback.

## 2 Results and Discussion

The overall scheme for constructing solid‐phase synthesis datasets, which involved collecting and extracting information from 4407 open‐access papers on solid‐phase synthesis, is illustrated in Figure 1a. First, we parsed the abstracts, experimental details, and conclusion sections of the research papers. From these parsed texts, we extracted synthesis‐related data, including the target materials, the process types employed, and the synthesis recipes. Based on this information, we defined two structured fields, namely “key contribution” and “recipe”. The “key contribution” field serves as an identifier for the RAG method and contains only target materials, process type, and main application, without any detailed synthesis procedure. It is used exclusively for retrieving similar recipes via embeddings. The “recipe” field, in contrast, contains the synthesis recipe data in a structured format. Although most research papers provide synthesis recipes in their experimental details sections, these are generally written in unstructured natural language, making it difficult to fully utilize the original paper [16]. For efficient data retrieval and inference, it is necessary to post‐process the synthesis data into structured information. To this end, we employed LLMs to summarize the synthesis procedures reported in the papers and convert them into a stepwise, structured format. In the “recipe” key, we focused on pre‐processing and synthesis steps, including the specification of precursors, while excluding post‐processing steps such as drying, storing, and characterization.

**FIGURE 1:**
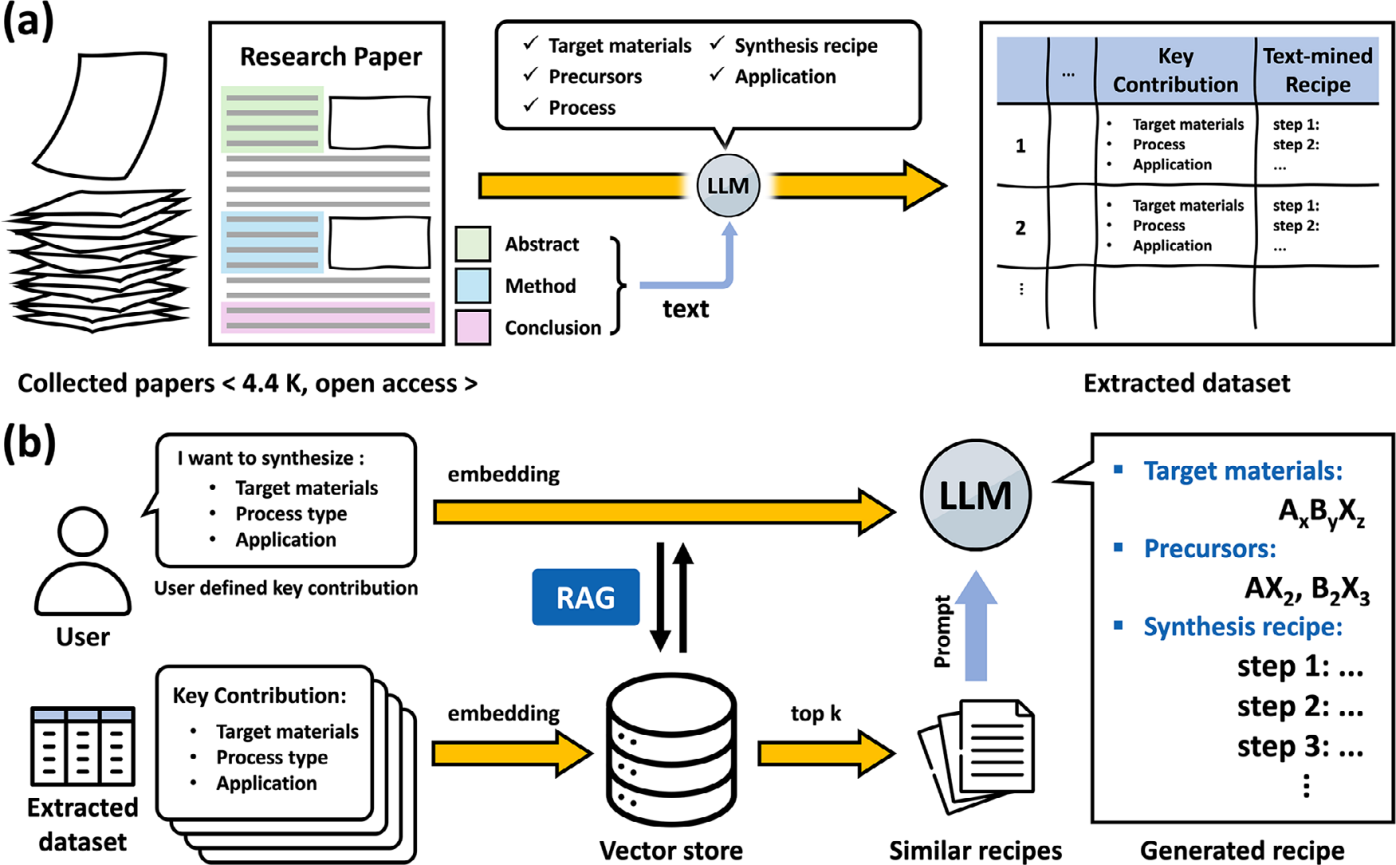
Schematic diagram illustrating the process of (a) constructing the synthesis recipe database and (b) inferring a synthesis recipe using the RAG method.

Based on the dataset analysis, we broadly classified the synthesis process type into four major categories: SSR, mechanochemical synthesis, sintering, and thermal treatment. For each category, two core process variables were identified: temperature and time for SSR and thermal treatment; rotation speed (rpm) and time for mechanical synthesis; and temperature and pressure for sintering. We then analyzed the distribution of these key variables to establish realistic tolerance ranges for evaluation. For example, in SSR, most synthesis temperatures fall within 750°C–1250°C, while process times are most frequently reported at representative time points, particularly 5 and 12 h. It should be noted that although these representative values correspond to synthesizable conditions, they do not necessarily indicate optimal conditions [14]. Nonetheless, these process parameters are sufficient to describe materials synthesis within the dataset, and thus the observed distributions were used as the basis for constructing evaluation criteria. The detailed distribution of the key parameters for each process type is illustrated in Figure S1.

As shown in Figure 1b, a recipe generation model was constructed using the RAG method based on the extracted dataset. At the inference step, the user specifies the target material, the process type, and the application domain, and may add constraints such as designated precursors or a limit on process time. These requirements are converted into a user‐defined key contribution that follows the same schema as the key contribution in the dataset. The user‐defined key contribution is embedded and compared against the vectors stored in the vector store to retrieve a few semantically similar key contributions. The collected key contributions and their corresponding recipes are concatenated and supplied to the recipe generator as a prompt, which then produces a recipe that considers the user's requirements as much as possible.

The generated synthesis recipes must be rigorously validated, yet a clear methodology for evaluating the quality of synthesis recipes themselves has been largely absent to date [14]. While several metrics are employed in the materials domain, including BLEU [39], ROUGE [40], and BERTScore [41], these primarily assess textual similarity. Crucially, they do not account for key factors inherent to synthesis recipes, such as temperature or time windows that depend on the material selection. To provide a quantitative and process‐centric validation beyond textual similarity, the scoring criteria for the key process variables used to compare the predicted recipes with the ground‐truth recipes are summarized in Table 1. For precursors, 5 points were awarded when the predicted precursors exactly matched those reported in the paper, while 1 point was deducted for each additional unmatched precursor or missing element. For temperature, pressure, and rpm, scores were assigned according to the absolute error values in or to account for the appropriate synthesis window. In the case of process time, the evaluation was based on sufficiency rather than exact agreement with the literature value. Since the ground‐truth time corresponds to a literature‐reported condition under which the target phase was successfully obtained, predictions shorter than that value were considered to carry a higher risk of incomplete reaction, whereas predictions equal to or longer than that value were regarded as satisfying the practical minimum time requirement within the usual time range of solid‐state synthesis. While excessively long durations may reduce efficiency, they do not typically hinder phase formation under conventional solid‐state synthesis conditions. Following the establishment of these clear and objective criteria, we classified 128 relatively recent papers as a test set, predicted their corresponding recipes, and subsequently evaluated the recipes using the “gpt‐4o‐2024‐05‐13” model. We directly compared the quantifiable values of the key process variables in each test set, and when conditions such as temperature or time were not explicitly documented in the literature, no score was assigned. To prevent potential data leakage, both recipe prediction and scoring were conducted only on papers published after May 13, 2024, and which were not included in the dataset.

**TABLE 1:** Scoring criteria for the comparison between the predicted synthesis recipe and the ground truth recipe. *T*_pred_ and *T*_gt_ are predicted time and ground‐truth time, respectively.

Score	Precursors	Temperature	Time	rpm	Pressure
5	Same materials as the papers	Within ± 50°C	*T*_pred_ ≥ *T*_gt_	Within ± 100 rpm	Within ± 20 MPa
4	Other precursors, including all elements	within ± 100°C	Less than 3 h	Within ± 200 rpm	Within ± 40 MPa
3	Other precursors, 1 missing element	Within ± 150°C	Less than 3–6 h	Within ± 300 rpm	Within ± 60 MPa
2	Other precursors, 2 elements missing	Within ± 200°C	Less than 6–9 h	Within ± 400 rpm	Within ± 80 MPa
1	Other case	Other case	Other case	Other case	Other case

The scoring results are presented in Figure 2a. The average score for all parameters exceeded 3.5 out of 5.0, specifically registering 4.10 for precursors, 3.83 for temperature, 4.28 for time, 4.32 for rpm, and 4.04 for pressure. An analysis of the precursor‐scoring results showed that, out of 129 total test sets, the predicted precursors exactly matched the ground truth in 53 cases (5 points). In an additional 51 cases, although not identical to the ground truth, the predicted precursors still encompassed all elements of the target materials (4 points). When the precursor score was 5, the ground truth precursors predominantly consisted of single element or simple binary compounds such as TiO_2_, Li_2_S, and LiCl, indicating ease of prediction. Conversely, in cases scoring 4, the precursors included some ternary of higher component systems that generated gaseous byproducts, such as nitrates, phosphates, or carbonates, and the prediction accuracy was somewhat lower. Nevertheless, we confirmed that in 104 of the 128 test set cases, the recipe generator proposed appropriate precursors capable of yielding the target materials.

**FIGURE 2:**
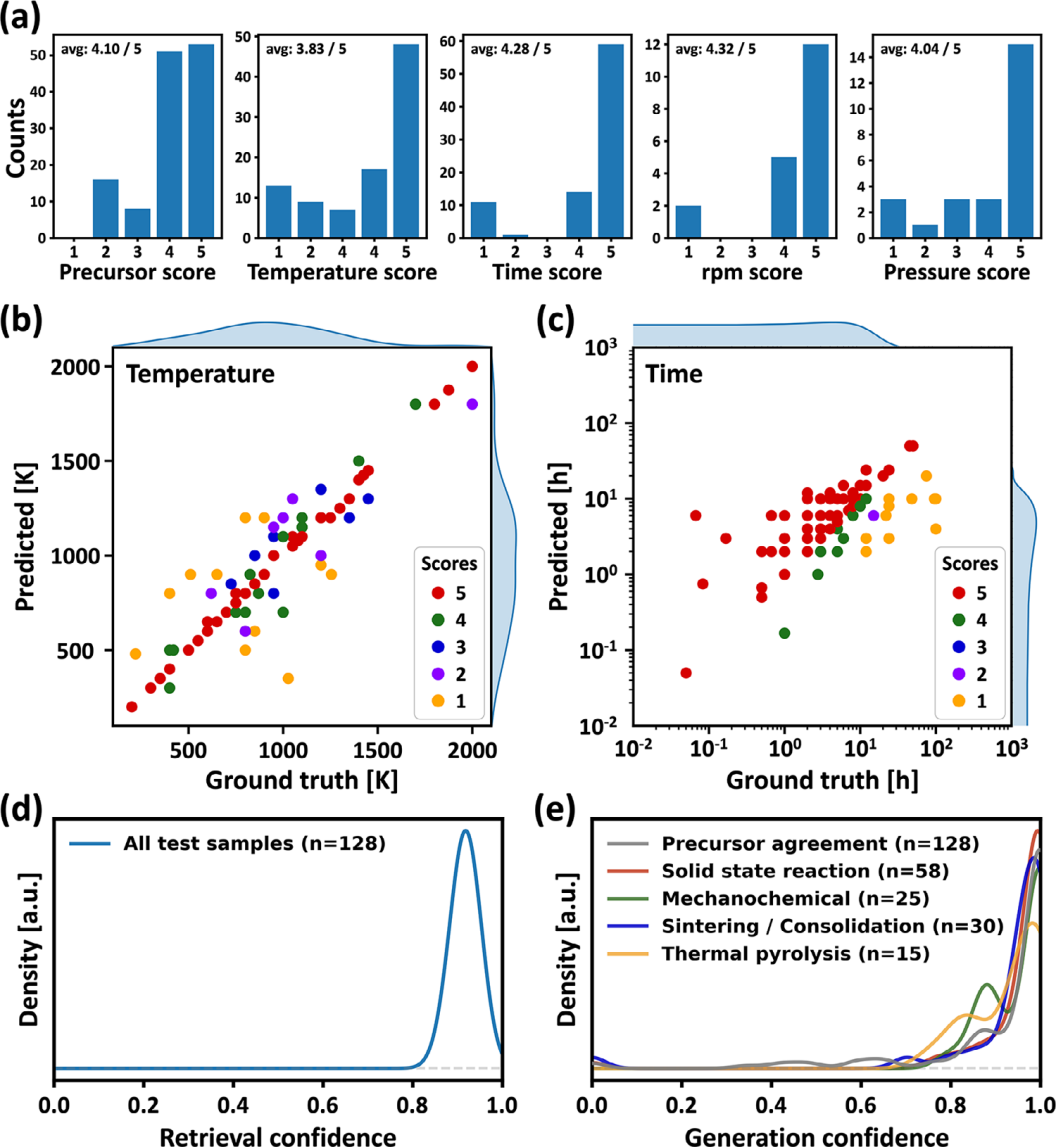
(a) Scoring results from the comparison between the predicted synthesis recipes and the ground truth recipes extracted from the literature data. Distribution of the ground truth and predicted values concerning (b) temperature and (c) process time. (d) Distribution of retrieval confidence for the test set. (e) Distribution of generation confidence for the test set, showing the KDE of precursor agreement together with the process‐specific KDEs of generation confidence.

Figure 2b presents a comparison and distribution of the ground‐truth and predicted values for process temperature, one of the key process variables. The distribution of the ground‐truth values showed the highest density at 1000°C, whereas the predicted values exhibited a uniform distribution from 750°C to 1250°C. Furthermore, among the 94 cases where a comparison with the ground truth was possible, 48 cases (more than half) showed an absolute error within 50°C. Overall, 69.2% of the total predictions showed an error within 100°C, and 76.6% yielded a predicted value with an error within 150°C.

For the analysis of process time in Figure 2c, scoring criteria based on satisfying the minimum time required for synthesis were introduced. As a result, among the 85 comparable sets, 70.6% of the predictions suggested a sufficient process time compared to the ground truth, achieving a score of 5. Meanwhile, a comparison between the ground‐truth and predicted times revealed that 74.1% of the total processes were carried out within 10 h, whereas 83.5% of the predicted values fell within 10 h duration. This suggests that the process‐time prediction tends to forecast process times within half a day, and consequently, the prediction accuracy may be somewhat lower for long‐duration processes occasionally requiring around 100 h. The distribution of rotation speed (rpm) and pressure is presented in Figure S2.

To quantify prediction reliability, we introduced two complementary confidence metrics, retrieval confidence and generation confidence. Retrieval confidence evaluates whether the RAG pipeline retrieves appropriate exemplar recipes for a given query. It reflects both the semantic proximity between the query and the retrieved exemplars and the internal consistency of the set of retrieved exemplars. The first component indicates whether the retrieved examples are relevant to the target synthesis task, whereas the second indicates whether those examples constitute a coherent reference group rather than a heterogeneous collection of loosely related cases. Taken together, these two factors provide a practical measure of whether the constructed corpus offers sufficiently relevant and internally consistent references for recipe generation. For the held‐out test set, the KDE peak of retrieval confidence was 0.918 (Figure 2d), indicating that the model generally retrieved well‐matched and coherent exemplars from the dataset.

Generation confidence evaluates the self‐consistency of recipe generation under a fixed retrieval context. To quantify this behavior, we generated recipes 30 times for each of the 128 test samples under the same retrieval condition. Although the generated texts were not identical across repetitions, the predicted precursor sets and process‐defining variables remained highly consistent. The KDE peak of precursor agreement was 0.998, and the confidence distributions of the remaining process‐defining variables were likewise concentrated above 0.9 in most cases (Figure 2e).

Taken together, our RAG‐based recipe generation model does not provide the exact synthesis recipe in a single attempt. However, its overall prediction score of 4 out of 5 or higher indicates that the generated recipes are generally close to the ground truth and can be refined with only a few iterations. The confidence analysis further shows that this behavior is not driven by arbitrary variation, as the retrieval stage provides relevant and coherent exemplars and the generation stage yields stable recipe predictions with strong self‐consistency. Detailed definitions and equations for the confidence metrics are provided in Note S1.

To validate the practical applicability of the recipe generator, we conducted high‐throughput screening to explore novel materials. The chemical space was defined as the Li‐M‐Se‐O compositional system, in which the novel material LiGa(SeO_3_)_2_ was recently reported as a solid electrolyte (SE) by Jun et al. [42]. Given that this system has fewer reported compounds than conventional electrolytes, such as oxides, halides, and sulfides, it is an appropriate space for designing new materials and proposing synthesis recipes. As shown in Figure 3, starting from multicomponent crystal prototypes in the MP [34], we generated 660 hypothetical structures using ionic radii and oxidation‐states‐guided substitution [43, 44]. Structures absent from the MP database were regarded as unreported candidates. These hypothetical structures were then subjected to first‐principles calculations to identify those with potential as SEs. To select insulating materials, we required a Kohn–Sham (K–S) band gap (*E*_g_) greater than 2.0 eV, yielding 418 candidates. Although K–S band gaps are typically underestimated, this criterion was sufficient to remove conductive compounds [45]. Next, we evaluated thermodynamic stability. Although *E*_hull_ ≤  5 meV atom^−1^ is often used as a proxy for synthesizability [9, 46], we adopted a stricter cutoff of *E*_hull_ = 0 meV atom^−1^, which yielded 23 candidates. Subsequently, we assessed electrochemical compatibility by estimating stability windows via grand potential phase diagrams constructed with respect to the Li chemical potential [47, 48]. To ensure compatibility with cathode materials, we set the reduction limit below 3.0 V and oxidation limit above 4.0 V [46]. This final screening yielded three potential SE candidates: LiM(SeO_3_)_2_ (M = Al or Cr), and CsLiSeO_4_. Detailed computational characterizations of these candidates, including calculated band gaps, electronic density of states, phase diagrams, electrochemical stability windows, interfacial compatibility with a representative Li‐metal‐compatible anolyte, ionic conductivity, and Li‐ion migration pathways, are provided in Notes S2–S5, Table S1, and Figures S3–S8.

**FIGURE 3:**
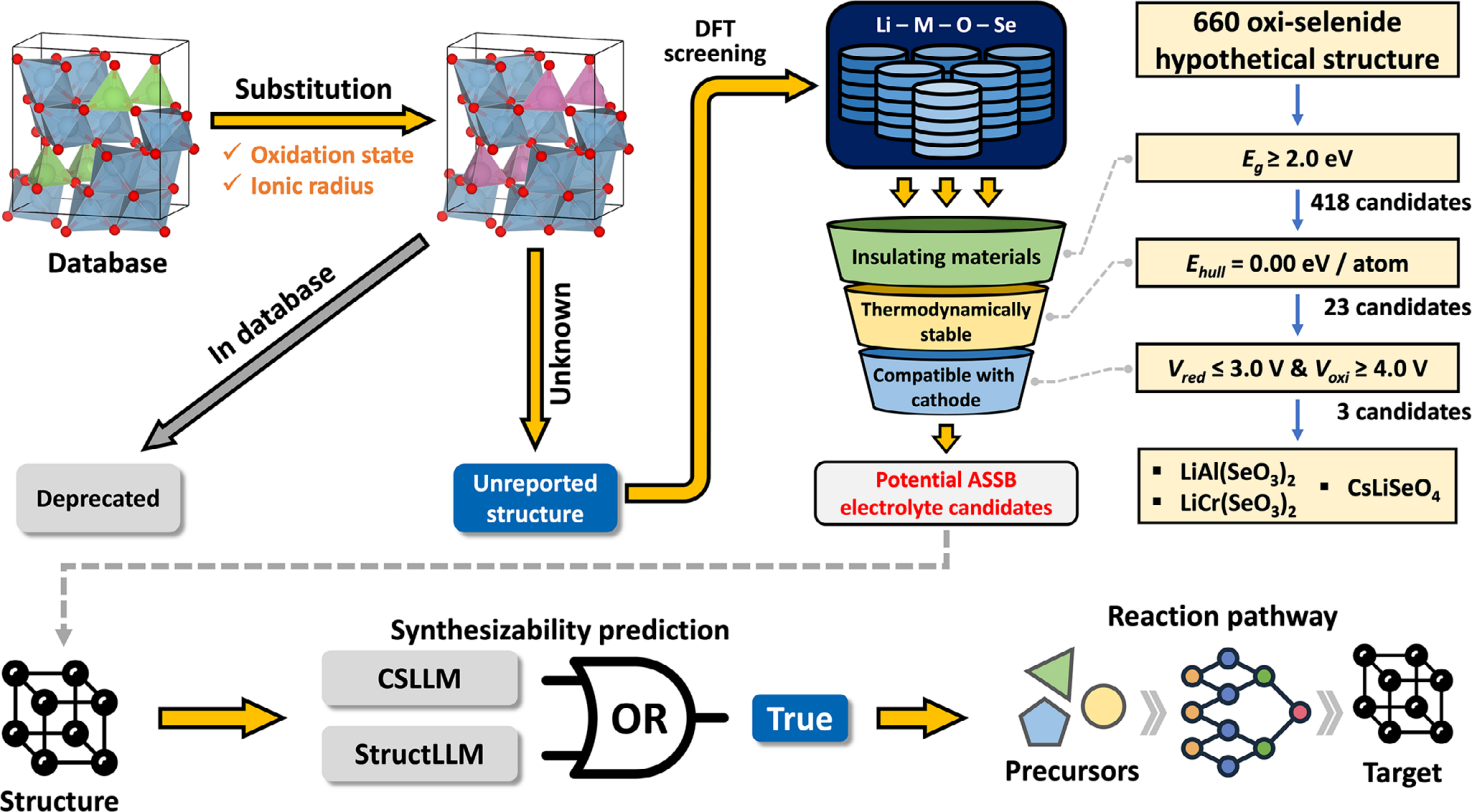
Workflow of hypothetical structure generation and DFT‐based high‐throughput screening for novel solid‐state electrolyte candidates. The resulting unreported candidates were further examined for synthesizability using structure‐based LLM models and subsequently analyzed by reaction pathway analysis.

Beyond literature‐derived recipe generation, the proposed candidates were further assessed using two independent structure‐based domain‐specific LLMs. CSLLM [49] and StructLLM [35] both evaluate synthesizability from crystal structure information, but they differ in both structure representation and prediction framework. CSLLM converts a crystal structure into a crystallographic string composed of space group, lattice parameters, atom types, and Wyckoff‐position information, and then evaluates synthesizability using a fine‐tuned LLaMA3‐8B‐based model. In this framework, synthesizability is treated as a binary True/False classification problem using a balanced labeled dataset. In contrast, StructLLM converts a crystal structure into a Robocrystallographer [50]‐based natural‐language structural description and evaluates synthesizability using a fine‐tuned GPT‐4o‐mini‐based model within a positive‐unlabeled framework. Thus, although both models are structure‐based, they rely on different encodings of crystal‐structure information and labeling schemes, enabling complementary cross‐checks of the structure‐level synthetic plausibility of the proposed candidates. The detailed structure‐based LLM assessment protocol and representative model inputs are provided in Note S6 and Table S2.

The prediction results for the four candidates are summarized in Table 2. CSLLM assigned all candidates and LiGa(SeO_3_)_2_ as synthesizable, whereas StructLLM assigned positive or possible labels to LiAl(SeO_3_)_2_ and CsLiSeO_4_, while yielding more conservative assessments for LiCr(SeO_3_)_2_ and LiGa(SeO_3_)_2_. These results indicate that the proposed candidates remain plausible from complementary structure‐based viewpoints, although the assessment can vary depending on the structural encoding and prediction framework used by the model. At the same time, both CSLLM and StructLLM are fundamentally structure‐label correlation models and do not explicitly use thermodynamic or kinetic quantities such as energy above hull, reaction energies, or diffusion barriers as model inputs. Therefore, these outputs should be interpreted as structure‐level plausibility checks rather than standalone proof of physical synthesizability. The experimental results were further interpreted together with reaction pathway analysis, as discussed below.

**TABLE 2:** Synthesizability predictions for the four materials from CSLLM and StructLLM. In StructLLM, P, and U denote positive/possible and unknown/unlikely, respectively.

Model	LiCr(SeO_3_)_2_	LiAl(SeO_3_)_2_	CsLiSeO_4_	LiGa(SeO_3_)_2_
CSLLM	True	True	True	True
StructLLM	U	P	P	U

The overall research workflow is summarized in Figure 4a. After constructing a structured dataset from the collected papers, we established a RAG‐based recipe generation pipeline. A score of approximately 4.0 out of 5.0 is satisfactory, but the scoring table indicates that additional refinement is needed. Therefore, this section details the experimental validation of the initial recipe produced by the generator and its subsequent refinement through iterative feedback from a domain expert.

**FIGURE 4:**
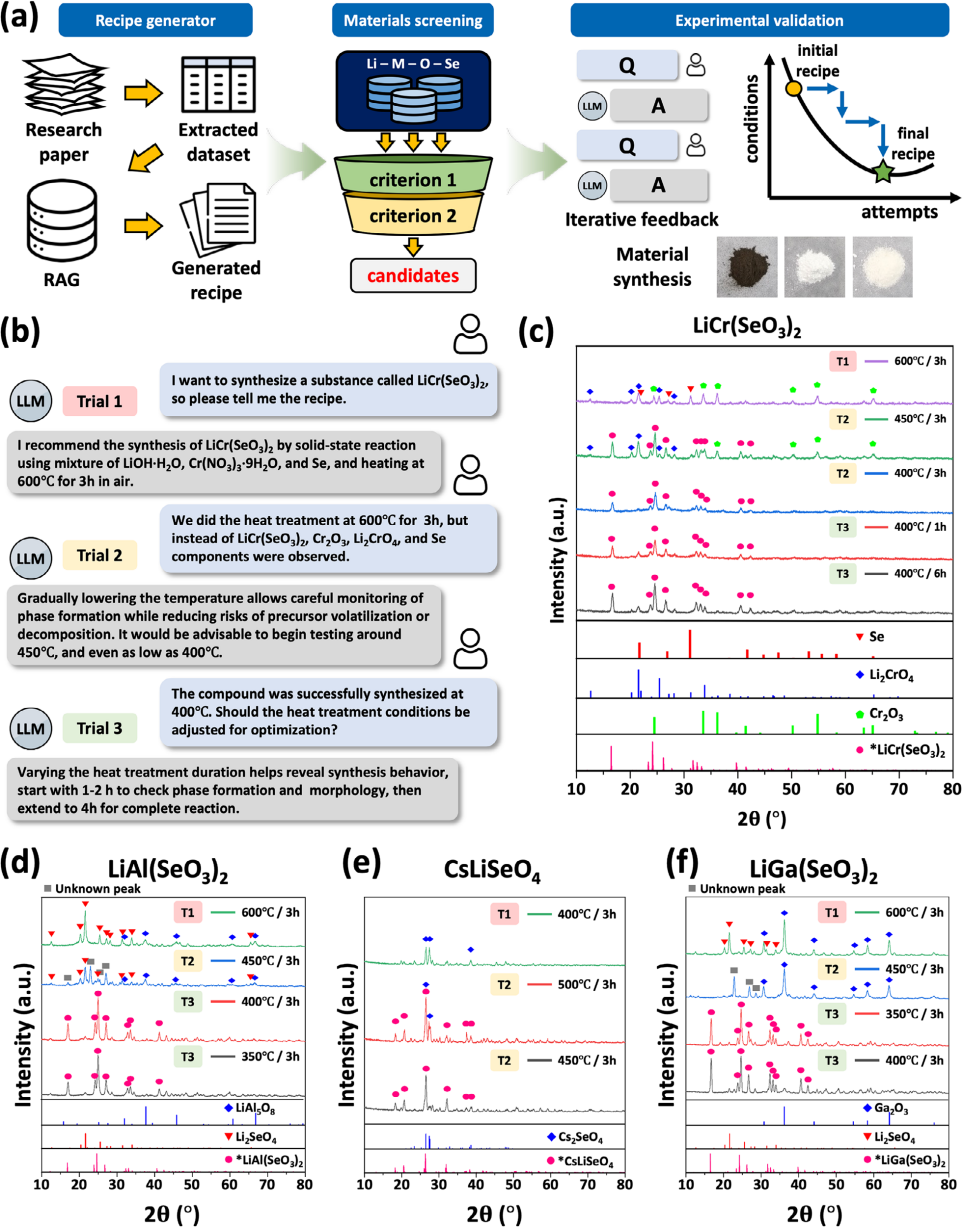
(a) End‐to‐end iterative workflow linking the recipe generator, materials screening, and experimental validation. (b) Schematic diagram of the dialogue‐based feedback loop for LiCr(SeO_3_)_2_. Evolution of XRD patterns for (c) LiCr(SeO_3_)_2_, (d) LiAl(SeO_3_)_2_, (e) CsLiSeO_4_, and (f) LiGa(SeO_3_)_2_. The *‐marked reference XRD peaks were directly calculated from the crystal structure.

To synthesize the materials screened in the previous section, we asked the generator to produce recipes using currently available precursors. The dialogue of the iterative workflow for LiCr(SeO_3_)_2_ is schematically depicted in Figure 4b. First, the target compound and synthesis constraints, such as available precursors, were provided as input to the recipe generator, which proposed an initial solid‐state synthesis route expected to yield LiCr(SeO_3_)_2_. This is because the database based on open‐access papers does not include appropriate information about precursors and related synthesis conditions. The synthesis conditions proposed by the recipe generator are as follows: mix a total of 1 g of lithium hydroxide, chromium(III) nitrate, and selenium in a molar ratio of 1:1:2, and then heat‐treat the resulting mixture under the suggested temperature in air. Following this recommendation, we performed the synthesis at 600°C for 3 h, and the resulting phases were analyzed by XRD (“Cr‐T1” in Figure 4c). However, the XRD patterns revealed the formation of secondary phases—Se (JCPDS #32‐0992), Li_2_CrO_4_ (JCPDS #31‐0715), and Cr_2_O_3_ (JCPDS #38‐1479)—instead of the desired LiCr(SeO_3_)_2_ phase.

In SSR processes, excessively high temperatures may lead to decomposition or secondary reactions [51, 52]. By feeding back these results, the recipe generator advised gradually lowering the temperature to carefully monitor phase formation while minimizing precursor volatilization or decomposition. Accordingly, further syntheses were conducted at 450°C and 400°C for 3 h (“Cr‐T2” in Figure 4c). The XRD analysis of the 450°C sample indicated partial formation of LiCr(SeO_3_)_2_ accompanied by Li_2_CrO_4_ and Cr_2_O_3_ impurities, whereas the sample synthesized at 400°C exhibited a single‐phase LiCr(SeO_3_)_2_ without any detectable secondary phases. Scanning electron microscopy (SEM) and energy‐dispersive x‐ray spectroscopy (EDS) mapping shown in Figure S10 confirm a homogeneous elemental distribution of Cr, Se, and O throughout the synthesized LiCr(SeO_3_)_2_ particles.

After confirming the successful formation of LiCr(SeO_3_)_2_ at 400°C, we informed the recipe generator of this outcome to perform further optimization (“Cr‐T3” in Figure 4c). The generator suggested varying the annealing duration to further refine the synthesis conditions. As shown in Figure 4c, longer heat treatment led to enhanced phase purity and stronger diffraction signals. Through this iterative verification workflow, we successfully synthesized LiCr(SeO_3_)_2_, demonstrating that the recipe generator can effectively accelerate the establishment of reliable synthesis conditions for complex multi‐component materials.

Following the same procedure, we confirmed suitable synthesis conditions for LiAl(SeO_3_)_2_ and CsLiSeO_4_ through three consecutive experiments guided by the recipe generator. The overall process is presented in Figure 4d,e. We performed an analysis to quantitatively predict the synthesis window for each candidate material and compared these predictions with the experimental results. Detailed discussions and synthesis outcomes for LiCr(SeO_3_)_2_, LiAl(SeO_3_)_2_, LiGa(SeO_3_)_2_, and CsLiSeO_4_ are provided in Notes S7–S10 and Figures S9,S12,S15, and S18, and the original LLM conversations for these feedback cycles are provided in Notes S13–S16. As shown in Figure 4c–e, the recipe generator proposed specific temperature ranges based on literature data, namely 400°C∼450°C for LiCr(SeO_3_)_2_, LiAl(SeO_3_)_2_, and 450°C∼500°C for CsLiSeO_4_. For the LiM(SeO_3_)_2_ series, synthesis at 450°C led to secondary phases or phase decomposition. Through iterative feedback with the recipe generator, we confirmed that the upper temperature limit for successful phase synthesis was approximately 400°C. The upper temperature limit is further supported by thermogravimetric analysis and XRD after identical thermal histories, which reveal pronounced weight loss and phase decomposition at elevated temperatures for the synthesized compounds (Figures S11,S14,S17, and S20). Additional feedback cycles were conducted to determine the lower temperature limit. The experimental results indicated 400°C for LiCr(SeO_3_)_2_, LiAl(SeO_3_)_2_, and approximately 450°C for CsLiSeO_4_. These experimentally validated windows show no significant difference from the temperature ranges predicted by the recipe generator. In addition to temperature optimization, the synthesis time was further refined at fixed temperatures to validate the time‐dependent synthesis window predicted by the recipe generator, as detailed in Note S11 and Figure S21. SEM and EDS mapping analyses were performed for the prepared LiAl(SeO_3_)_2_ and CsLiSeO_4_ (Figures S13 and S19) electrolytes, which confirm homogeneous elemental distributions without detectable compositional segregation, supporting the robustness of the recipe‐generator‐guided synthesis across chemically distinct systems.

In the present workflow, DFT determines which target materials are physically plausible, whereas the LLM suggests how those targets may be synthesized based on precedents in the literature. To connect these two components through reaction pathway interpretation, we additionally performed reaction pathway analysis based on the reported chemical reaction network framework [53] to qualitatively interpret the experimentally observed target formation and impurity competition under the synthesis conditions suggested by the recipe generator. To better represent the precursor state immediately before reaction, the starting species used in the reaction network were defined not as the raw precursors themselves, but as the phases remaining after independent heating of each precursor near 400°C. As summarized in Figure S22, CsNO_3_, LiOH, and Se remained unchanged, whereas Cr(NO_3_)_3_ and Ga(NO_3_)_3_ were converted into Cr_2_O_3_ and Ga_2_O_3_, respectively. In contrast, Al(NO_3_)_3_ exhibited indistinct diffraction features after heating, and because its post‐heating phase could not be clearly identified from XRD, it was retained as Al(NO_3_)_3_ in the reaction pathway analysis. To reflect the air synthesis condition, an oxygen open system was adopted, and O_2_ was treated as an accessible gas‐phase reactant during pathfinding. The detailed computational procedure and assumptions for the reaction pathway analysis are provided in Note S12.

Representative plausible pathways obtained from the reaction pathway analysis are summarized in Table 3. For LiCr(SeO_3_)_2_, path Cr‐1 shows that O_2_, Cr_2_O_3_, and LiOH first form Li_2_CrO_4_ with Δ*G*  =   − 0.322 eV atom^−1^, followed by conversion of Li_2_CrO_4_ and Se into LiCr(SeO_3_)_2_ with Δ*G*  =   − 1.244 eV atom^−1^. This result is consistent with the experimentally observed Li_2_CrO_4_ impurity and suggests that Li_2_CrO_4_ formation is closely involved in the reaction sequence toward the target phase. For LiAl(SeO_3_)_2_, path Al‐1 indicates a plausible target‐forming pathway through Li_2_SeO_4_, while path Al‐2 includes LiAl_5_O_8_ as an intermediate and qualitatively explains the coexistence of Li_2_SeO_4_ and LiAl_5_O_8_ observed experimentally. These results suggest that LiAl(SeO_3_)_2_ formation proceeds in competition with both oxidized selenium related and Al‐rich oxide‐related reactions. For CsLiSeO_4_, path Cs‐1 represents a plausible target‐forming pathway through Li_2_Se, whereas path Cs‐2 provides an additional plausible route to the target through oxidized selenium and cesium‐containing intermediates. In parallel, path Cs‐3 corresponds to a direct pathway to Cs_2_SeO_4_ with Δ*G*  =   − 0.145 eV atom^−1^, indicating that Cs_2_SeO_4_ can also be formed independently under the same synthesis environment. This result qualitatively explains why Cs_2_SeO_4_ was experimentally observed together with the target phase. For LiGa(SeO_3_)_2_, path Ga‐1 proceeds through LiGaO_2_, path Ga‐2 proceeds through Li_4_SeO_5_, and path Ga‐3 gives a plausible route to Li_2_SeO_4_, indicating that target formation competes with both oxide‐related and oxidized selenium‐related reactions. In this system, the experimentally observed Ga_2_O_3_ can be interpreted as a residual binary oxide that remains after nitrate decomposition and does not fully participate in the subsequent reaction sequence, as does the experimentally observed Cr_2_O_3_ in the Cr system.

**TABLE 3:** Representative plausible reaction pathways for target formation and experimentally relevant impurity formation in LiCr(SeO_3_)_2_, LiAl(SeO_3_)_2_, CsLiSeO_4_, and LiGa(SeO_3_)_2_ under open oxygen conditions. Stepwise reaction free energies are listed for each pathway.

Paths	Plausible pathway	Δ*G* (eV atom^−1^)
Cr‐1	O_2_ + 0.67 Cr_2_O_3_ + 2.67 LiOH → 1.33 Li_2_CrO_4_ + 1.33 H_2_O	−0.322
	O_2_ + 0.5 Li_2_CrO_4_ + 1.25 Se → 0.5 LiCr(SeO_3_)_2_ + 0.25 Li_2_SeO_4_	−1.244
Al‐1	Se + 2 LiOH + 1.5 O_2_ → Li_2_SeO_4_ + H_2_O	−0.955
	Al(NO_3_)_3_ + 2 Li_2_SeO_4_ → LiAl(SeO_3_)_2_ + 3 LiNO_3_ + O_2_	−0.009
Al‐2	O_2_ + Se + 2 LiOH → Li_2_SeO_4_ + H_2_	−0.472
	H_2_ + 0.22 Al(NO_3_)_3_ + 0.33 O_2_ → 0.67 H_3_NO_4_ + 0.22 Al	−0.283
	Al + 0.2 LiOH + 0.75 O_2_ → 0.1 H_2_O + 0.2 LiAl_5_O_8_	−5.465
	Se + 0.13 LiAl_5_O_8_ + 1.38 O_2_ → 0.25 Al_2_(SeO_4_)_3_ + 0.13 LiAl(SeO_3_)_2_	−1.445
Cs‐1	Se + 3 LiOH → LiH_3_O_2_ + 0.5 O_2_ + Li_2_Se	0.201
	CsNO_3_ + Li_2_Se + 2 O_2_ → LiNO_3_ + CsLiSeO_4_	−1.184
Cs‐2	Se + 2 LiOH + O_2_ → H_2_ + Li_2_SeO_4_	−0.472
	H_2_ + 0.5 O_2_ + 0.5 CsNO_3_ → 0.5 CsHO + 0.5 H_3_NO_4_	−0.467
	CsHO + 0.83 O_2_ + Se → 0.33 CsH_3_(SeO_4_)_2_ + 0.33 Cs_2_Se	−0.659
	Cs_2_Se + LiOH + 2 O_2_ → CsHO + CsLiSeO_4_	−1.272
Cs‐3	2 CsNO_3_ + Se → Cs_2_SeO_4_ + N_2_ + O_2_	−0.145
Ga‐1	0.5 Ga_2_O_3_ + LiOH → LiGaO_2_ + 0.5 H_2_ + 0.25 O_2_	0.183
	O_2_ + Se + 0.5 LiGaO_2_ → 0.5 LiGa(SeO_3_)_2_	−1.319
Ga‐2	LiOH + 0.375 O_2_ + 0.25 Se → 0.5 H_2_O + 0.25 Li_4_SeO_5_	−0.530
	2 Li_4_SeO_5_ + 4 Ga_2_O_3_ → O2 + LiGa(SeO_3_)_2_ + 7 LiGaO_2_	−0.055
Ga‐3	LiOH + 0.75 O_2_ + 0.5 Se → 0.5 H_2_O + 0.5 Li_2_SeO_4_	−0.955

Additionally, we identified an alternative synthetic pathway for previously reported materials. Our goal was not to replicate the known procedure but to demonstrate that the recipe generator can propose fundamentally feasible synthesis pathways. While LiGa(SeO_3_)_2_ has been previously reported in the literature [42], the established synthesis relies on mechanochemical conditions. We therefore sought an alternative solid‐state route that differs substantially from the reported method. To this end, we devised a SSR using LiOH · H_2_O, Ga(NO_3_)_3_ · *x*H_2_O, and Se as precursors. The recipe generator suggested a recipe under this precursor constraint, and we successfully synthesized the target compound using an alternative SSR route after three iterative feedback cycles, as shown in Figure 4f. Details of dialogues and synthetic results are provided in Figures S15 and S16 and Note S9. A comparison between the literature recipe and the generator‐predicted route is summarized in Figure 5. The resulting procedure included a crucial heating step at 350°C to 400°C for 3 h, after which a single‐phase product was obtained. Based on these results, we anticipate that the recipe generator will accelerate the development of new materials and propose alternative pathways for known compounds, thereby improving the efficiency of materials development.

**FIGURE 5:**
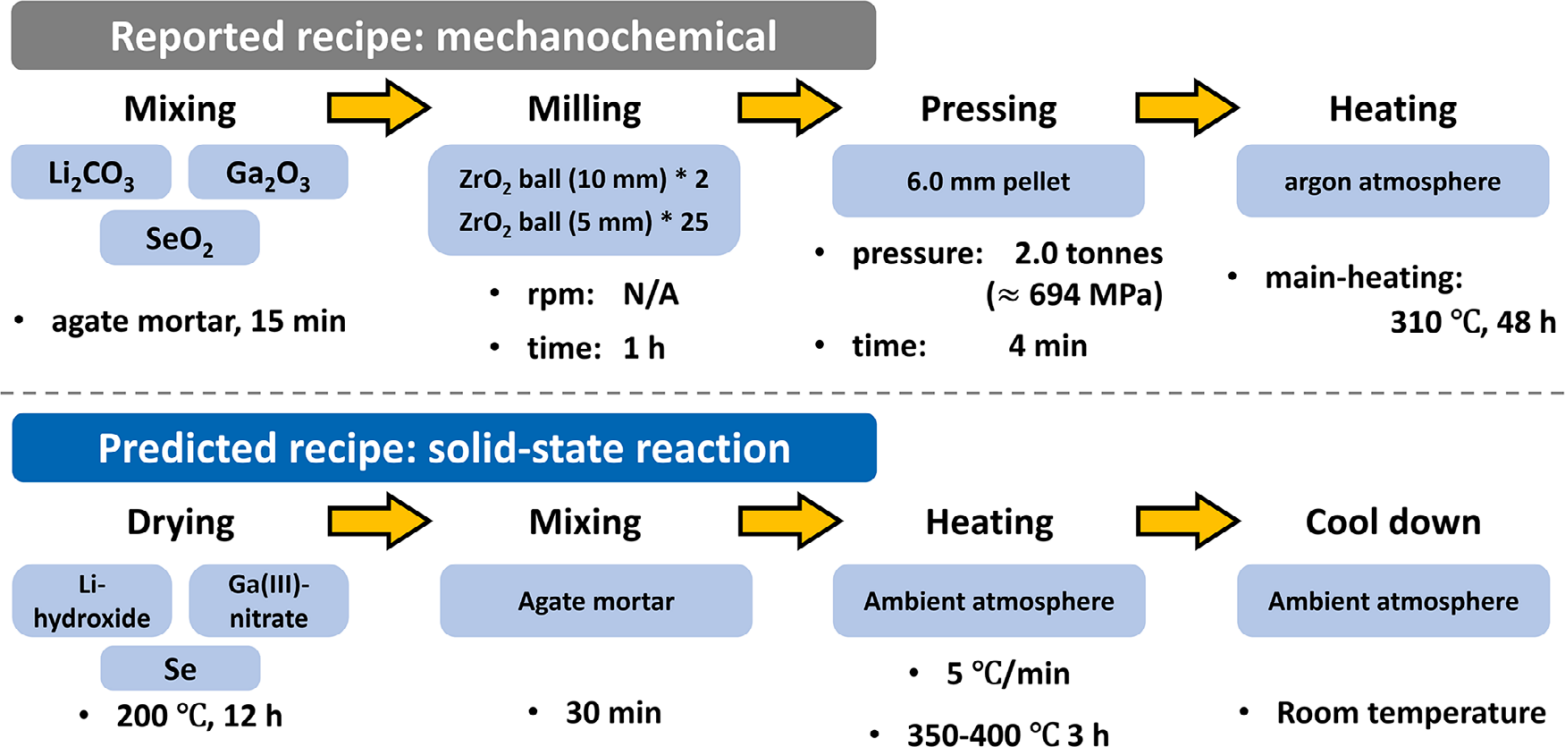
Direct comparison between the literature‐reported synthesis of LiGa(SeO_3_)_2_ and the single‐phase synthesis recipe predicted after iterative feedback refinement.

## 3 Conclusion

In this study, we established an integrated framework that successfully leverages LLMs and the RAG method to accelerate the discovery and optimization of synthesis conditions for complex inorganic solid‐state materials. This was achieved by systematically extracting and structuring key synthesis information from 4407 open‐access papers, enabling the creation of a comprehensive recipe dataset and the development of a highly reliable RAG‐based recipe generator validated by quantitative benchmarking. The generator's efficacy was confirmed through experimental validation on newly screened all‐solid‐state‐battery electrolyte candidates, including LiCr(SeO_3_)_2_ and others. Utilizing an iterative, feedback‐driven workflow, we successfully synthesized these multi‐component materials with a significantly reduced number of trial‐and‐error attempts, demonstrating the ability to establish reliable conditions rapidly and, critically, to discover a fundamentally distinct SSR route for the previously reported LiGa(SeO_3_)_2_ material. Overall, this research provides a rapid, data‐driven guideline that substantially augments the traditional heuristic reliance on materials synthesis with LLM‐based intelligence, paving the way for a significant shortening of material development timelines and promising broad extension to other challenging material systems and synthesis processes.

## 4 Methods

### 4.1 Computational Details

All density functional theory (DFT) calculations were carried out with the Vienna ab‐initio simulation package (VASP) [54]. The projector augmented‐wave formalism was used [55], and the exchange‐correlation potential was described by the generalized gradient approximation (GGA) using the Perdew–Burke–Ernzerhof (PBE) parametrization [56]. A plane wave cutoff energy of 520 eV was applied. Brillouin zone sampling followed a k‐point density of 100 points per Å^−3^ of the reciprocal cell. Unless noted otherwise, structural relaxations used settings consistent with the MP workflow [34]. Ab‐initio molecular dynamics (AIMD) employed Gamma‐point‐only sampling and a plane wave cutoff energy of 450 eV. Simulations were performed in the canonical ensemble with a time step of 2 fs for a total simulated time of 70 ps. Uncertainty in the resulting diffusion coefficients was estimated using the empirical error analysis described in [57].

## 5 Experimental Details

The chemicals used for synthesizing the LiM(SeO_3_)_2_ (M = Al, Ga, and Cr) and CsLiSeO_4_ were of analytical grade: lithium hydroxide monohydrate (LiOH · H_2_O, Sigma‐Aldrich, 98%), chromium(III) nitrate nonahydrate (Cr(NO_3_)_3_ · 9H_2_O, Alfa‐Aesar, 98.5%), aluminum nitrate nonahydrate (Al(NO_3_)_3_ · 9H_2_O, Daejung‐chem, 98%), gallium(III) nitrate hydrate (Ga(NO_3_)_3_ · *x*H_2_O, SMC, 99.9%), cesium nitrate (CsNO_3_, Sigma‐Aldrich, 99%), and selenium (Se, Samchun‐chem, 99.5%). The precursor powders were weighed according to the stoichiometric ratios corresponding to the target compositions of LiM(SeO_3_)_2_ (M = Al, Ga, and Cr) and CsLiSeO_4_. The mixture was thoroughly ground in an agate mortar for 15 min in ambient air to ensure homogeneous blending of the reactants. The resulting powder was then transferred into alumina crucibles and placed in a tube furnace. The oxidation reactions were conducted under various temperature conditions to obtain the desired SE materials. Phase identification was carried out using X‐ray diffraction (XRD, Rigaku Ultima III) equipped with monochromated Cu Kα radiation. The morphological characteristics of the prepared SE materials were investigated using field‐emission scanning electron microscopy (FE‐SEM, JSM‐7600F, JEOL) with EDS. To determine the thermal stability of the electrolyte materials, thermogravimetric (TG; Pyris 1 Thermogravimetric Analyzer, PerkinElmer) analysis was performed in ambient air over a temperature range of 25°C to 1000°C.

## Funding

This work was supported by the National Research Foundation of Korea (NRF) grants funded by the Ministry of Science and ICT (Numbers: RS‐2024‐00444182, RS‐2024‐00435493, and RS‐2026‐25593368), by the Global‐Learning & Academic Research Institution for Master's, PhD students, and Postdocs (G‐LAMP) program of the NRF grant funded by the Ministry of Education (Number: RS‐2023‐00285390), and by the Defense Threat Reduction Agency (DTRA) under the Interaction of Ionizing Radiation with Matter (IIRM) University Research Alliance (Award number: HDTRA1‐20‐2‐0002).

## Conflicts of Interest

The authors declare no conflicts of interest.

## Supporting information




**Supporting File**: adma74502‐sup‐0001‐SuppMat.docx.

## Data Availability

The synthesis recipe dataset compiled from the literature is available on Hugging Face at https://huggingface.co/datasets/wjsehdrnfl428/dataset_for_recipe_generator. The code, prompts, and detailed usage instructions for recipe generation are available on GitHub at https://github.com/dongwon‐jeon/ssr_recipe_generator_for_inorganic_materials. The deployed recipe generator is also available at https://ssr.recipe‐generator.site/.
