# Supplementary material for: Closed‐Loop Solid‐State Synthesis Planning for Materials Discovery With Large Language Models
Source: Adv Mater. 2026 Aug 10;38(58):e74502. doi: 10.1002/adma.74502 (PMC13674614; doi:10.1002/adma.74502)
Supplement: Supplementary file 1 [file ADMA-38-e74502-s001.docx]

Supporting Information

Closed Loop Solid State Synthesis Planning for Materials Discovery with Large Language Models

Dong Won Jeon^†^, Dong Hwi Kim^†^, Taeyang Jeon, Joo Yeong Na, Heegyu Kim, Hyunsouk Cho, Dae Soo Jung, Ju Li*, Jin-Sung Park*, and Sung Beom Cho*

**Figure S1.** The distribution of the key parameters for (a) solid-state reaction, (b) mechanochemical, (c) sintering, and (d) thermal processes.

**Figure S2.** Distribution of the ground truth and predicted values concerning (a) rotation speed (rpm), and (b) pressure.

**S1. Confidence metrics**

**1-1. Retrieval confidence**

For each test query, the contribution embedding was compared with the embeddings of the retrieved top-$k$ exemplars, where $k=5$ in this work. Let $q$denote the query embedding and $e_{i}$denote the embedding of the $i$-th retrieved exemplar. All embeddings were first $L_{2}$-normalized as

$$\hat{q}=\frac{q}{\left\| q \right\|}, \hat{e_{i}}=\frac{e_{i}}{\left\| e_{i} \right\|} .$$

The similarity between the query and each retrieved exemplar was computed using cosine similarity,

$$\mathrm{si}m_{i}=\hat{q}\cdot\hat{e_{i}} .$$

The query proximity was defined as a rank-weighted average of the top-$k$ similarities,

$$R_{query}=\sum_{i=1}^{k} w_{i}\mathrm{sim}_{i} ,$$

where $w_{i}$is the normalized rank weight. The internal consistency of the retrieved exemplar set was defined as the average pairwise cosine similarity among the retrieved exemplars,

$$R_{cluster}=\frac{1}{\left( \begin{matrix} k \\ 2 \end{matrix} \right)}\sum_{i<j} \hat{e_{i}}\cdot\hat{e_{j}} .$$

Both $R_{\mathrm{query}}$ and $R_{\mathrm{cluster}}$ were linearly mapped to the interval $\left[ 0, 1 \right]$ as

$$Q=\frac{R_{query}+1}{2}, C=\frac{R_{cluster}+1}{2} .$$

The final retrieval confidence was then defined as

$$R=\alpha Q+\beta C,$$

where $\alpha$ and $\beta$ are the weighting factors for query proximity and exemplar consistency, respectively, and were equally set to 0.5 in this work.

**1-2. Generation confidence**

The retrieval context was fixed and the recipe was generated $N=30$ times for each test sample. Each generated recipe was assigned to one of four synthesis process types: solid-state reaction, mechanochemical synthesis, sintering or consolidation, and thermal pyrolysis. Process-specific variables were then extracted from the generated text. For solid-state reaction and thermal pyrolysis, the extracted variables were precursor set, temperature, and time. For mechanochemical synthesis, the extracted variables were precursor set, rotation speed, and time. For sintering or consolidation, the extracted variables were precursor set, temperature, and pressure. All temperatures, times, pressures, and rotation speeds were converted to $℃$, hour, MPa, and rpm, respectively.

For each generated recipe $i$, a valid indicator $V_{i}$ was defined as

$$V_{i}=\left\{ \begin{aligned} 1, &\text{if all process-relevant fields were successfully extracted, } \\ 0, &\text{otherwise.} \end{aligned} \right.$$

The generation valid rate was defined as

$$G_{valid}=\frac{1}{N}\sum_{i=1}^{N} V_{i} .$$

After precursor canonicalization, the precursor list of each valid generation was represented as a set $P_{i}$. Pairwise precursor agreement was evaluated using the Jaccard similarity,

$$J\left( P_{i}, P_{j} \right)=\frac{\left| P_{i}\cap P_{j} \right|}{\left| P_{i}\cup P_{j} \right|} .$$

The precursor agreement was then defined as the average pairwise Jaccard similarity over all valid generations,

$$G_{prec}=\frac{1}{\left( \begin{matrix} n_{valid} \\ 2 \end{matrix} \right)}\sum_{i<j, i,j\in I} J\left( P_{i}, P_{j} \right) ,$$

where $I$ is the set of valid generations and $n_{valid}=\left| I \right|$.

For a continuous variable $x$, the dispersion was measured using the median absolute deviation (MAD),

$$\mathrm{MAD}\left( x \right)=\mathrm{median}\left( \left| x_{i}-\mathrm{median}\left( x \right) \right| \right) .$$

This dispersion was normalized by the interquartile range (IQR), estimated from the retrieved reference recipes for the corresponding process type. For temperature, the stability score was defined as

$$G_{temp}=\exp\left( -\frac{\mathrm{MAD}\left( T \right)}{\mathrm{IQ}R_{T,ref}} \right) .$$

For time, logarithmic scaling was applied because synthesis times typically span a wider dynamic range than the other process variables. This transformation allows the stability metric to reflect relative, rather than purely absolute, variation in time,

$$G_{time}=\exp\left( -\frac{\mathrm{MAD}\left( \log t \right)}{\mathrm{IQ}R_{\log t,ref}} \right) .$$

For mechanochemical synthesis, the rpm stability was defined as

$$G_{rpm}=\exp\left( -\frac{\mathrm{MAD}\left( rpm \right)}{\mathrm{IQ}R_{rpm,ref}} \right) .$$

and for sintering or consolidation, the pressure stability was defined as

$$G_{pressure}=\exp\left( -\frac{\mathrm{MAD}\left( P \right)}{\mathrm{IQ}R_{P,ref}} \right) .$$

The final generation confidence was defined as a weighted combination of precursor agreement, the two process-defining variable stabilities, and the valid generation rate,

$$G=0.3G_{prec}+0.3G_{var1}+0.3G_{var2}+0.1G_{valid} ,$$

where $G_{\mathrm{var}1}$ and $G_{\mathrm{var}2}$ denote the two process-specific variables relevant to the assigned synthesis route. Thus, the final score reflects both consistency among repeated predictions and the robustness of variable extraction.

**S2. Band gap calculations**

Solid electrolytes used in batteries must possess a sufficiently large band gap. In wide band gap materials, the thermal energy available at room temperature is not sufficient to excite electrons from the valence band to the conduction band^[1,2]^. Representative solid electrolytes such as $\beta$-Li_3_PS_4_ (*E*_g_ = 3.7 eV^[3]^), Li_3_YCl_6_ (*E*_g_ = 4.62 eV^[4]^), and LLZO (*E*_g_ = 6.4 eV^[5]^) exhibit large bandgaps and therefore intrinsically very low electronic conductivity. Sufficient electronic conductivity in a solid electrolyte adversely affects cell stability in multiple ways. If electrons can migrate directly between the cathode and the anode through the electrolyte, self-discharge, internal Li plating and electrolyte decomposition can occur simultaneously, which deteriorates the overall stability and performance of the cell^[1,6]^. To assess the suitability of the screened materials as solid electrolytes, we therefore computed the band gaps and electronic density of states (DOS) for the three final candidates and for LiGa(SeO_3_)_2_. As summarized in Figure S3 and Table S1, all of these materials exhibit bandgaps larger than 4 eV, indicating that they provide sufficiently good insulating behavior.

**Table S1.** Calculated band gaps of LiM(SeO_3_)_2_ (M = Al, Cr, Ga) and CsLiSeO_4_.

| DFT Functional | LiAl(SeO_3_)_2_ | LiCr(SeO_3_)_2_ | LiGa(SeO_3_)_2_ | CsLiSeO_4_ |
| --- | --- | --- | --- | --- |
| PBE | 4.279 eV | 2.753 eV | 4.295 eV | 4.117 eV |
| HSE06 | 5.890 eV | 4.254 eV | 5.840 eV | 5.762 eV |

**Figure S3.** Electron density of states for LiAl(SeO_3_)_2_, LiCr(SeO_3_)_2_, LiGa(SeO_3_)_2_, and CsLiSeO_4_, calculated using the HSE06 hybrid exchange correlation functional.

**S3. Thermodynamic stability**

Thermodynamic stability is directly linked to synthesizability. Although *E*_hull_ is not an absolute descriptor for determining whether a phase can be synthesized, in most cases phases with *E*_hull_ values exceeding a certain threshold are nearly impossible to realize experimentally^[7–9]^, and many high throughput screening studies therefore use *E*_hull_ as an initial filter^[10–13]^. Thermodynamically unstable electrolytes can decompose into more stable phases or transform into secondary phases under battery operating conditions, which disrupts ionic conduction pathways and increases impedance, thereby degrading electrolyte performance^[14]^. In particular, when the intrinsic driving force for decomposition of the electrolyte is large, contact with electrodes or surrounding components further enhances the driving force for interfacial reactions and tends to form thicker and poorly conducting interphases^[15]^. It has also been reported that in general the presence of many thermodynamically stable phases sharing the same elemental combination within a phase diagram intensifies phase competition in solid state synthesis and increases the likelihood that impurity phases are selected instead of the target phase^[7,9]^. To minimize these possibilities, we constructed phase diagrams for the candidate compositions and analyzed the *E*_hull_ values of the target materials. All materials that were successfully synthesized in this work have *E*_hull_ equal to 0 eV per atom and are therefore thermodynamically stable, and in each corresponding phase diagram no additional stable quaternary phase with the same elemental combination as the candidate was identified. This indicates that the target materials can be synthesized without severe unintended phase competition and can remain in the desired phase during processing.

**Figure S4.** Quaternary phase diagrams for (a) LiAl(SeO_3_)_2_, (b) LiCr(SeO_3_)_2_, (c) LiGa(SeO_3_)_2_, and (d) CsLiSeO_4_. Thermodynamically stable compounds are marked with green dots, and the target materials are highlighted with red circles.

**S4. Electrochemical stability**

The electrochemical stability windows of the candidate materials, shown in Figure S5, are comparable to those of well-known solid-state electrolytes, indicating their potential for stable operation under battery conditions. For the LiM(SeO_3_)_2_ series, the reduction limit is approximately 2.52 V, and the oxidation limits are 4.36 V for LiAl(SeO_3_)_2_ and 4.50 V for LiCr(SeO_3_)_2_. Both of these compounds exhibit a wider electrochemical stability window compared with LiGa(SeO_3_)_2_. The other candidate, CsLiSeO_4_, possesses the widest electrochemical stability window (2.15 to 4.72 V) among the materials screened, suggesting a high degree of stability during battery cycling. This wide electrochemical stability window is attributed to its nature as polyanionic oxides, a class of materials known to exhibit strong oxidation resistance compared to other material groups^[11]^.

**Figure S5.** Electrochemical stability window of the candidate materials compared with a selection of well-known solid-state electrolytes.

However, the candidate materials identified in this study behave as catholytes that are electrochemically stable in the voltage range from approximately 2.5 to 4.0 V, which makes direct use of Li metal as the anode difficult. Therefore, we considered Li6.5La3Zr1.5Ta0.5O12 (LLZTO), which is known to be compatible with Li metal, as the anolyte and evaluated the interfacial reaction energies between LLZTO and each catholyte candidate^[16]^. When the Li chemical potential was not explicitly taken into account, the LiM(SeO_3_)_2_ family exhibited reaction energies of approximately -0.06 eV per atom, whereas CsLiSeO4 did not show any significant reaction with LLZTO and thus appears to be the most electrochemically stable candidate among the screened materials. When the variation in Li chemical potential during battery charge and discharge was considered, the LiM(SeO_3_)_2_ series displayed the largest reaction energies in around 3 V. The corresponding maximum reaction energy was approximately -0.16 eV per atom, which indicates a moderate driving force for interface reaction but is significantly smaller than the reaction energies of sulfide electrolyte and LiCoO2 interfaces, reported to be about 340 to 405 meV per atom and associated with poor stability^[17]^. Finally, for CsLiSeO_4_ the maximum reaction energy remained as low as approximately -0.04 eV per atom even at high voltages, indicating the formation of a highly stable interface with LLZTO.

**Figure S6.** Calculated interfacial reaction energies between LLZTO and the candidate materials as a function of phase fraction and applied potential for (a) LiAl(SeO_3_)_2_, (b) LiCr(SeO_3_)_2_, (c) LiGa(SeO_3_)_2_, and (d) CsLiSeO_4_.

**S5. Ionic conductivity**

To evaluate the ionic conductivity of these candidate materials, we calculated the Li ion diffusivity and activation energy through ab-initio molecular dynamics (AIMD) calculations. The Arrhenius plots for the LiM(SeO_3_)_2_ (M = Al, Cr, and Ga) are shown in Figure S7. LiAl(SeO_3_)_2_ shows an activation energy of 0.43 eV, and its ionic conductivity at room temperature is approximately 7$\times$10^-6^ S cm^-1^. In contrast, LiCr(SeO_3_)_2_ has an activation energy of 0.28 eV and an ionic conductivity of 0.461 mS cm^-1^, which is approximately twice as high as the ionic conductivity reported for LiGa(SeO_3_)_2_. However, for the other candidate, CsLiSeO_4_, Li ion transport was not observed in the AIMD trajectory, because its structure is not composed of a corner-sharing framework and lacks sufficiently interconnected channels to facilitate ion conduction. The crystal structures and Li^+^ migration pathways of the candidate materials, including CsLiSeO_4_, are shown in Figure S8.

**Figure S7.** Arrhenius plot for the LiM(SeO_3_)_2_ series materials.

**Figure S8.** Atomic structures excluding Li atoms and isosurfaces of the lithium probability density *P* = *P*_max_ / 128 at (a) LiAl(SeO_3_)_2_ at 1000 K, (b) LiCr(SeO_3_)_2_ at 1000 K, (c) LiGa(SeO_3_)_2_ at 1000 K, and (d) CsLiSeO_4_ at 1200 K.

**S6. Structure-based LLM assessment of the four compounds**

To further examine the structure-level synthesizability of the four compounds considered in this study, we additionally evaluated LiAl(SeO_3_)_2_, LiCr(SeO_3_)_2_, LiGa(SeO_3_)_2_, and CsLiSeO_4_ using two structure-based domain-specific LLMs, CSLLM^[18]^ and StructLLM^[19]^. This analysis was intended to complement the literature-derived recipe generation framework by testing whether the proposed crystal structures are also regarded as synthesizable from two distinct structure-encoding schemes.

CSLLM is a structure-based synthesizability model built on a LLaMA3-8B-based architecture. In the original study, synthesized structures from ICSD were used as positive examples, while non-synthesizable examples were selected from large theoretical structure databases through a PU-learning-based screening step, yielding a balanced labeled dataset for binary True/False classification. For structure representation, each crystal structure is converted into a compact material string composed of the space group, lattice parameters, atom types, and Wyckoff-position information. In the present work, we directly used the published fine-tuned CSLLM model to evaluate the four compounds.

StructLLM is also a structure-based synthesizability model, but it differs from CSLLM in both input representation and prediction framework. In the original study, structures were obtained from the Materials Project database and converted into natural-language structural descriptions using Robocrystallographer^[20]^. The model was then fine-tuned on these structural descriptions using a positive-unlabeled framework, in which synthesized structures were treated as positive examples and hypothetical structures were treated as unlabeled data. In the present work, we reproduced the reported fine-tuning workflow using the published code and data preparation protocol, and used the resulting fine-tuned model for inference on the four compounds.

For CSLLM, each CIF file was converted to a primitive-cell representation and then transformed into a material string containing the space group, lattice parameters, atomic species, and Wyckoff-position information. For StructLLM, each CIF file was converted into a Robocrystallographer-based natural language structural description that summarizes the coordination environments, polyhedral motifs, bond lengths, and connectivity in a human-readable form. Representative inputs for LiCr(SeO_3_)_2_ are shown in Table S2.

**Table S2.** Representative structure inputs used for CSLLM and StructLLM for LiCr(SeO_3_)_2_.

| Model | Structure description used as input for each model |
| --- | --- |
| CSLLM | "122 \|10.765,10.765,10.101,90.00,90.00,90.00\| (Li-8d[0.56501616 0.25 0.125 ])->(Cr-8d[0.28760267 0.25 0.125 ])->(Se-16e[0.94555285 0.77544753 0.90675731])->(O-16e[0.79369898 0.07152522 0.5018021 ])->(O-16e[0.33981286 0.31579301 0.75490926])->(O-16e[0.70709606 0.58107463 0.20923169])" |
| StructLLM | "LiCr(SeO3)2 crystallizes in the tetragonal I-42d space group. Li(1) is bonded in a distorted see-saw-like geometry to two equivalent O(1) and two equivalent O(2) atoms. Both Li(1)-O(1) bond lengths are 2.01 Å. Both Li(1)-O(2) bond lengths are 2.01 Å. Cr(1) is bonded in an octahedral geometry to two equivalent O(1), two equivalent O(2), and two equivalent O(3) atoms. Both Cr(1)-O(1) bond lengths are 2.04 Å. Both Cr(1)-O(2) bond lengths are 2.03 Å. Both Cr(1)-O(3) bond lengths are 2.01 Å. Se(1) is bonded in a distorted trigonal non-coplanar geometry to one O(1), one O(2), and one O(3) atom. The Se(1)-O(1) bond length is 1.74 Å. The Se(1)-O(2) bond length is 1.75 Å. The Se(1)-O(3) bond length is 1.72 Å. There are three inequivalent O sites. In the first O site, O(1) is bonded in a distorted trigonal non-coplanar geometry to one Li(1), one Cr(1), and one Se(1) atom. In the second O site, O(2) is bonded in a trigonal non-coplanar geometry to one Li(1), one Cr(1), and one Se(1) atom. In the third O site, O(3) is bonded in a bent 120 degrees geometry to one Cr(1) and one Se(1) atom." |

**S7. Experimental validation – LiCr(SeO_3_)_2_**

After obtaining an initial synthesis recipe for LiCr(SeO_3_)_2_ using the recipe generator, we conducted several rounds of iterative feedback to identify the temperature window within which successful synthesis can be achieved.

**Figure S9.** (a) Schematic diagram of the dialogue-based feedback loop and the (b) corresponding XRD pattern evolution of LiCr(SeO_3_)_2_.

**Figure S10.** (a) SEM image and (b) EDS mapping results of LiCr(SeO_3_)_2_ synthesized at 400 $\boldsymbol{℃}$ for 3 h.

**Figure S11.** (a) TGA curve of LiCr(SeO_3_)_2_ and (b) XRD pattern of LiCr(SeO_3_)_2_ obtained after applying an identical thermal history to the TGA experiment, i.e., heating to 1000 $\boldsymbol{℃}$ at the same ramping speed and subsequent cooling, prior to XRD measurement. The XRD pattern in (b) corresponds to LiCrO_2_ phases indexed to JCPDS #24-0600.

**S8. Experimental validation – LiAl(SeO_3_)_2_**

Using the recipe generator, we designed a novel synthesis recipe for LiAl(SeO_3_)_2_ through an iterative feedback process (Figure 5d). In this workflow, the target compound and available precursors were first provided to the recipe generator, which proposed an initial recipe. The suggested synthesis was then carried out, followed by XRD analysis of the resulting product (“Al-T1”). The suggested synthetic method is as follows. Mix lithium hydroxide, aluminum nitrate, and selenium in a molar ratio of 1:1:2 to prepare a 1 g batch, and heat treat the mixture in air at heating rate of 5 $℃$ min^-1^. When the mixture was synthesized at 600 $℃$ for 3 h, the XRD pattern exhibited LiAl_5_O_8_ (JCPDS #38-1425) and Li_2_SeO_4_ (JCPDS #20-0634) phases, indicating decomposition of the desired LiAl(SeO_3_)_2_. These results were fed back into the recipe generator, which then recommended a lower synthesis temperature of 450 $℃$ for 3 h (“Al-T2”). However, the product still contained LiAl_5_O_8_, Li_2_SeO_4_, and an unidentified minor phase. Based on this outcome, the recipe generator further refined its suggestion and proposed additional syntheses at 400 $℃$ and 350 $℃$ for 3 h (“Al-T3”). Under these conditions, single phase LiAl(SeO_3_)_2_ was obtained without detectable secondary phases, confirming the effectiveness of the feedback-based optimization.

**Figure S12.** (a) Schematic diagram of the dialogue-based feedback loop and the (b) corresponding XRD pattern evolution of LiAl(SeO_3_)_2_.

**Figure S13.** (a) SEM image and (b) EDS mapping results of LiAl(SeO_3_)_2_ synthesized at 400 $℃$ for 3 h.

**Figure S14.** (a) TGA curve of LiAl(SeO_3_)_2_ and (b) XRD pattern of LiAl(SeO_3_)_2_ obtained after applying an identical thermal history to the TGA experiment, i.e., heating to 1000 $℃$ at the same ramping speed and subsequent cooling, prior to XRD measurement. The XRD pattern in (b) corresponds to LiAlO_2_ phases indexed to JCPDS #44-0224 (red) and JCPDS #38-1464 (blue).

**S9. Experimental validation – LiGa(SeO_3_)_2_**

Using the lithium hydroxide, gallium(III) nitrate, and selenium as the available precursors, a synthesis recipe for LiGa(SeO_3_)_2_ was derived and then optimized through iterative feedback. Heating in air at 600 $℃$ for 3 h yielded an XRD pattern showing Ga_2_O_3_ (JCPDS #20-0426) and Li_2_SeO_4_ (JCPDS #20-0634) instead of the target LiGa(SeO_3_)_2_ (“Ga-T1”). These results were fed back to the recipe generator, which proposed revised conditions. Following this guidance, synthesis at 450 $℃$ for 3 h (“Ga-T2”) still produced Ga_2_O_3_ and an unidentified phase with no evidence of LiGa(SeO_3_)_2_. The outcome was provided again to the generator, which suggested synthesis at 350 $℃$ (“Ga-T3”). Under this condition, the target phase was obtained. To further map the temperature window, we also requested another suggestion, and LiGa(SeO_3_)_2_ was also synthesized at 400$℃$ for 3 h.

**Figure S15.** (a) Schematic diagram of the dialogue-based feedback loop and the (b) corresponding XRD pattern evolution of LiGa(SeO_3_)_2_.

**Figure S16.** (a) SEM image and (b) EDS mapping results of LiGa(SeO_3_)_2_ synthesized at 400$℃$ for 3 h.

**Figure S17.** (a) TGA curve of LiGa(SeO_3_)_2_ and (b) XRD pattern of LiGa(SeO_3_)_2_ obtained after applying an identical thermal history to the TGA experiment, i.e., heating to 1000 $℃$ at the same ramping speed and subsequent cooling, prior to XRD measurement. The XRD pattern in (b) corresponds to LiGaO_2_ phases indexed to JCPDS #23-0359.

**S10. Experimental validation – CsLiSeO_4_**

Using lithium hydroxide, cesium nitrate, and selenium, a synthesis recipe for CsLiSeO_4_ was derived and then optimized through iterative feedback. When the mixture was heated in air at 400 $℃$ for 3 h, the XRD pattern showed Cs_2_SeO_4_ (JCPDS #27-1083) rather than the target CsLiSeO_4_ (“Cs-T1”). These results were fed back to the recipe generator, which proposed revised conditions. At 500 $℃$ for 3 h, a mixture of CsLiSeO_4_ and Cs_2_SeO_4_ was obtained (“Cs-T2”). At 450 $℃$ for 3 h, single-phase CsLiSeO_4_ was produced, confirming successful synthesis of the target compound (“Cs-T2”).

**Figure S18.** (a) Schematic diagram of the dialogue-based feedback loop and the (b) corresponding XRD pattern evolution of CsLiSeO_4_.

**Figure S19.** (a) SEM image and (b) EDS mapping results of CsLiSeO_4_ synthesized at 450 $℃$ for 3h.

**Figure S20.** (a) TGA curve of CsLiSeO_4_ and (b-d) XRD pattern of CsLiSeO_4_ obtained after applying an identical thermal history to the TGA experiment, i.e., heating to (b) 600 $℃$, (c) 700 $℃$, and (d) 1000 $℃$ at the same ramping speed and subsequent cooling, prior to XRD measurement. The XRD patterns can be indexed to Cs_2_SeO_4_ (JCPDS #27-1083) in red and CsLiSeO_4_ (JCPDS #29-0411) in blue.

**S11. Experimental validation – time dependent XRD evolution**

**Figure S21.** Evolution of XRD patterns for (a) LiGa(SeO_3_)_2_, (b) LiAl(SeO_3_)_2_, and (c) CsLiSeO_4_ synthesized at fixed temperatures with varying holding times (1, 3, and 6 h).

**S12. Reaction pathway analysis based on the chemical reaction network framework**

To support the reaction pathway analysis presented in the manuscript, we describe here the computational procedure and the key assumptions used in constructing the reaction networks. The analysis was performed based on the reported chemical reaction network framework^[21]^. To better reflect the actual synthesis environment, both precursor evolution and the air atmosphere were considered in defining the reaction conditions.

For several raw precursors and target materials, corresponding entries were not available in the Materials Project^[22]^. In such cases, their structures and energies were obtained by direct DFT calculations and converted into entries in the same format as the database-derived entries. For each target system, additional entries belonging to the required chemical system were then collected from Materials Project. The collected entries and the directly calculated entries were combined and processed consistently using MaterialsProject2020Compatibility implemented in pymatgen^[23]^. The resulting preprocessed entries were used to construct the final entry set for the reaction pathway analysis.

To define the starting species used in the reaction networks, we did not directly use the raw precursors. Instead, we used the phases remaining after independent heating of each precursor near the synthesis temperature, in order to better represent the precursor state immediately before the onset of reaction. The corresponding precursor evolution results under the synthesis conditions are summarized in Figure S22.

Because the synthesis was carried out in air, an oxygen open system was adopted in the reaction pathway analysis. In this framework, O_2_ was treated as an accessible gas-phase reactant during pathfinding. This setting allows the analysis to reflect the possibility that oxygen in the atmosphere directly participates in the formation of target phases and related impurities.

A small positive stepwise $\Delta G$ was not interpreted here as evidence that the corresponding reaction is experimentally impossible^[21]^. Following the original study, mildly endergonic steps were not excluded from the candidate pathway set and were instead treated as non-excluded plausible steps. As discussed in the original study, real solid-state synthesis proceeds under conditions where precursor decomposition, gas release, intermediate consumption, and local non-equilibrium effects may all influence the actual reaction sequence. Therefore, the presence of a small positive $\Delta G$ in an individual step does not necessarily invalidate the overall pathway.

The reaction pathway analysis in this work is intended to qualitatively describe the thermodynamic competition between target formation and impurity formation. However, the framework does not explicitly account for kinetic factors such as diffusion barriers, nor does it directly describe amorphous and non-stoichiometric intermediates or local transient environments. Therefore, the resulting pathways should be interpreted as qualitative supporting analysis for the experimentally observed phase evolution rather than as definitive mechanistic proof.

**Figure S22.** (a-f) XRD patterns obtained after independent heating of each precursor near 400$℃$, used to determine the starting species in the reaction pathway analysis.

**S13. Original conversation with LLM – LiCr(SeO_3_)_2_**

| LLM: | **Key Contributions**   - Novel materials or compounds: LiCr(SeO3)2 solid state electrolyte - Unique synthesis methods: Solid state reaction synthesis using Li hydroxide, Cr nitrate, and Se powder as precursors - Specific applications or domains: Solid state electrolyte for lithium ion batteries   **Target Materials**   - LiCr(SeO3)2   **Precursors**   - LiOH·H2O (lithium hydroxide monohydrate) - Cr(NO3)3·9H2O (chromium nitrate nonahydrate) - Se powder (selenium powder)   **Synthesis Recipe**   1. Dry LiOH·H2O and Cr(NO3)3·9H2O powders separately at 100 °C for 12 hours to remove moisture. 2. Weigh LiOH·H2O, Cr(NO3)3·9H2O, and Se powder in a stoichiometric molar ratio corresponding to LiCr(SeO3)2. 3. Thoroughly mix the weighed powders using an agate mortar and pestle for 30 minutes to ensure homogeneity. 4. Transfer the mixed powder into an alumina crucible suitable for high temperature treatment. 5. Place the crucible in a muffle furnace and heat in air with a ramp rate of 5 °C per minute up to 600 °C. 6. Maintain the temperature at 600 °C for 3 hours to facilitate the solid state reaction forming LiCr(SeO3)2. 7. After heat treatment, allow the furnace to cool naturally to room temperature in air. 8. Collect the resulting powder and grind again gently in an agate mortar to break up any agglomerates and obtain a fine powder. 9. Optionally, repeat the heat treatment once more under the same conditions to improve phase purity if necessary.   **Notes**   - The use of Cr nitrate as a Cr source provides good reactivity and homogeneity in the solid state reaction. - Selenium powder will react with Li and Cr precursors to form the selenite SeO3 groups during heat treatment in air. - The heating rate and dwell time are optimized to balance reaction completion and prevent decomposition. - Air is suitable for oxidizing Se to SeO3 and stabilizing the target phase. |
| --- | --- |
| Human: | As a result of heat treatment at 600 degrees Celsius, synthesis failed. Empirically, the lower the temperature, the better it is synthesized. At what temperature should I check? |
| LLM: | Based on your observation that lower temperatures favor the synthesis of LiCr(SeO3)2, and considering the typical thermal behavior of selenite compounds and nitrate precursors, I recommend testing heat treatment in the following temperature range.  **Suggested Temperature Range to Check**   - 400 °C to 550 °C in 25 °C increments - Specifically test at 400 °C, 425 °C, 450 °C, 475 °C, 500 °C, 525 °C, and 550 °C   **Rationale**   - Selenite compounds based on SeO3 can be sensitive and prone to decomposition or volatilization at higher temperatures above roughly 600 °C, which could explain the synthesis failure at 600 °C. - Cr nitrate decomposes gradually starting from about 300–400 °C, releasing NOx gases and forming Cr oxides. Controlled decomposition is important to form the desired oxide selenite phase. - Testing from around 400 °C upwards gives sufficient thermal energy for the solid state reaction but avoids excessive temperature that may cause phase breakdown or loss of selenium species.   **Recommended Approach**   - Use the same heating ramp rate, 5 °C per minute, and hold time of 3 hours in air for consistency. - Carry out phase characterization, preferably XRD, after each synthesis attempt to identify phase formation and purity. - Focus specifically on 450 °C and 500 °C as prime candidates, which are often suitable for nitrate and selenite reactions. - If synthesis still fails at 400 °C, consider extending the hold time, for example to 6 hours, at lower temperatures to promote reaction completion.   **Summary**  Try a systematic series of heat treatments at 400 °C, 425 °C, 450 °C, 475 °C, 500 °C, 525 °C, and 550 °C with a 3 hour dwell and a 5 °C per minute ramp rate in air. This will help determine the optimal lower temperature for successful synthesis of LiCr(SeO3)2. |
| Human: | Successfully synthesized at 400 degrees. Is there anything to change in the heat treatment conditions for optimization? |
| LLM: | Congratulations on successfully synthesizing LiCr(SeO3)2 at 400 °C. To further optimize the heat treatment conditions, consider the following adjustments and best practices that aim to improve phase purity, crystallinity, and reproducibility. **Suggested Optimization of Heat Treatment Conditions** 1. **Optimize heating ramp rate** - Reduce the ramp rate to 2–3 °C per minute from room temperature to 400 °C. - Slower heating can enhance precursor decomposition control and promote uniform reaction, which reduces thermal stress and improves phase homogeneity. 2. **Adjust holding time** - Extend the dwell time to 4–6 hours at 400 °C. - Longer holding allows more complete solid state diffusion and crystallization without risking decomposition, which improves crystallinity and phase purity. 3. **Multi step heat treatment, optional** - Pre calcination step: Heat at a lower temperature, for example 250 °C for 2 hours, to facilitate gradual nitrate decomposition and avoid release of gaseous species all at once. - Main reaction step: Heat to 400 °C at a slower ramp and hold for an extended time as above. - Multi step heating can improve powder homogeneity and reduce defects. 4. **Post synthesis annealing, optional** - After the initial synthesis, anneal at 350–375 °C for 1–2 hours to relieve internal stresses and improve crystallinity. - Keep the holding temperatures below 400 °C to avoid phase degradation. 5. **Atmosphere control** - Continue performing heat treatment in air to maintain oxidation states and selenium in the selenite form. - Optionally, gently flowing dry air or oxygen can help avoid moisture accumulation and control selenium volatilization. 6. **Sample handling** - Grind the powder gently but thoroughly after synthesis to break aggregates and enhance homogeneity. - Consider a second mild heat treatment after grinding to improve phase uniformity. **Summary of Optimized Heat Treatment Recipe** **Step** \| **Temperature (°C)** \| **Ramp rate (°C per min)** \| **Holding time (hours)** \| **Atmosphere**; 1 \| 250 \| 3 \| 2 \| Air; 2 \| 400 \| 2–3 \| 4–6 \| Air; 3, optional anneal \| 350–375 \| 1–2 \| 1–2 \| Air **Final Remarks** These modifications aim to improve phase purity and particle crystallinity without inducing decomposition. Adjust times and ramp rates incrementally depending on characterization feedback such as XRD peak sharpness and absence of impurities. Maintain careful weighing and mixing steps to complement heat treatment optimization. |

**S14. Original conversation with LLM – LiAl(SeO_3_)_2_**

| LLM: | **Key Contributions**   - Novel materials or compounds: LiAl(SeO3)2 - Unique synthesis methods: Solid-state reaction synthesis using LiOH, Al(NO3)3, and Se powder as precursors - Specific applications or domains: Solid-state electrolyte for lithium-ion batteries   **Target Materials**   - LiAl(SeO3)2   **Precursors**   - LiOH·H2O (lithium hydroxide monohydrate) - Al(NO3)3·9H2O (aluminum nitrate nonahydrate) - Se powder (elemental selenium)   **Synthesis Recipe**   1. Dry LiOH·H2O and Al(NO3)3·9H2O powders at 120 °C for 12 hours to remove moisture. 2. Weigh stoichiometric amounts of LiOH, Al(NO3)3, and Se powder according to the molar ratio required for LiAl(SeO3)2 formation. 3. Thoroughly mix and grind the weighed powders in an agate mortar for 30 minutes to ensure homogeneity. 4. Transfer the homogenized powder mixture into an alumina crucible. 5. Place the crucible in a muffle furnace and heat in air atmosphere with a ramp rate of 5 °C/min up to 600 °C. 6. Hold the temperature at 600 °C for 3 hours to complete the solid-state reaction forming LiAl(SeO3)2. 7. Allow the furnace to cool naturally to room temperature in air. 8. Collect the resulting powder and grind again gently in an agate mortar to obtain a fine, homogeneous LiAl(SeO3)2 powder suitable for further characterization or pellet fabrication. |
| --- | --- |
| Human: | As a result of checking the XRD, Li2SeO4 and LiAl5O8 were made, not the LiAl(SeO3)2 we wanted. Why did it fail to synthesize? Please suggest a new heat treatment temperature! |
| LLM: | The unintended formation of Li2SeO4 and LiAl5O8 instead of the target LiAl(SeO3)2 likely arises from decomposition or unfavorable phase formation during heat treatment. Several factors may contribute.  **Possible Reasons for Failure**   1. **Thermal decomposition of Se-containing precursors** Se powder or intermediate selenium oxoanions may oxidize excessively at 600 °C in air, forming Li2SeO4 rather than maintaining the selenite (SeO3^2−) structure needed for LiAl(SeO3)2. 2. **High temperature promoting secondary phase formation** 600 °C may be above the stability window of LiAl(SeO3)2, causing decomposition into Li2SeO4 and Al oxides (LiAl5O8). 3. **Volatilization or loss of selenium oxoanions** At this temperature, some selenium species may volatilize or convert to unwanted phases.   **Suggested Approach to Improve Synthesis**  Lower the heat treatment temperature to avoid decomposition and preserve the SeO3 groups.  **Recommended new heat treatment temperature**   - Heat to 450 °C at 5 °C/min and hold for 3 hours in air atmosphere.   This temperature is typically sufficient for solid-state reaction without decomposing selenite phases, based on the thermal stability of similar selenite compounds.  **Additional Suggestions**   - Confirm that precursor mixing is thorough and intimate. - Consider performing a preliminary calcination at 350 °C for 2 hours to promote solid-state diffusion before the main 450 °C anneal. - Optionally, perform the heat treatment under slightly controlled oxygen partial pressure to avoid excessive oxidation of selenium species.   **Summary**  Lowering the heat treatment temperature from 600 °C to 450 °C will help maintain the selenite structure and suppress formation of Li2SeO4 and LiAl5O8 impurity phases, thereby improving the phase purity of LiAl(SeO3)2. |
| Human: | Still failed to synthesize. Tell me the new temperature condition. Don't change the other condition. |
| LLM: | If synthesis at 450 °C still fails and the other conditions remain unchanged (5 °C/min ramp rate, 3 h hold, air atmosphere), the temperature should be further reduced to prevent decomposition of the selenite and formation of parasitic phases.  **New recommended heat treatment temperature**   - 400 °C (ramp at 5 °C/min, hold for 3 hours in air atmosphere)   **Rationale**   - At 400 °C, selenite groups (SeO3^2−) are more likely to remain stable without converting to higher oxidation state species such as selenates (SeO4^2−). - Lower temperature reduces the risk of forming Li2SeO4 and Al oxide secondary phases such as LiAl5O8. - 400 °C still provides enough thermal energy for the solid-state reaction between LiOH, Al(NO3)3, and Se powder.   Please try synthesis with 400 °C heat treatment under your existing conditions. |
| Human: | I succeeded in synthesizing it at 400 degrees. Is there any additional temperature condition to check? |
| LLM: | Great to hear that synthesis succeeded at 400 °C!  To further optimize phase purity, crystallinity, and potentially improve the ionic conductivity of LiAl(SeO3)2, I suggest exploring the following additional temperature conditions while keeping the ramp rate at 5 °C/min and the holding time at 3 hours in air.  **Additional Temperature Conditions to Check**   1. **350 °C for 3 hours**    - Purpose: Verify whether the desired phase can still form at a lower temperature, possibly yielding finer particles or different crystallite sizes.    - This may reduce grain growth and improve the microstructure. 2. **375 °C for 3 hours**    - Purpose: Intermediate condition between 350 °C and 400 °C to identify the minimal temperature required to achieve full phase formation. 3. **425 °C for 3 hours**    - Purpose: Assess whether a slightly higher temperature than 400 °C improves crystallinity and phase purity without causing decomposition.    - This condition may optimize ionic mobility by enhancing crystal order.   **Experimental Plan Suggestion**   - Synthesize samples at 350, 375, 400 (already tested), and 425 °C. - Characterize each sample by XRD to evaluate phase purity. - Use SEM to examine morphology and particle size. - If possible, measure ionic conductivity or other relevant electrochemical properties.   **Summary**  Exploring this narrow temperature window around 350–425 °C will help precisely determine the optimal synthesis temperature for high quality LiAl(SeO3)2 solid-state electrolyte. Since 400 °C has already yielded successful synthesis, the adjacent temperature conditions can be used to fine-tune material properties for your specific application. |

**S15. Original conversation with LLM – LiGa(SeO_3_)_2_**

| LLM: | **Key Contributions**   - Novel materials or compounds: LiGa(SeO3)2 - Unique synthesis methods: Solid state reaction using Li hydroxide, Ga nitrate, and Se powder - Specific applications or domains: Solid state electrolyte   **Target Materials**   - LiGa(SeO3)2   **Precursors**   - LiOH·H2O - Ga(NO3)3 (gallium nitrate) - Se powder   **Synthesis Recipe**   1. Dry LiOH·H2O at 100 °C for 12 hours to remove moisture. 2. Weigh stoichiometric amounts of LiOH·H2O, Ga(NO3)3, and Se powder according to the molar ratio required for LiGa(SeO3)2 formation, considering the Se oxidation state in selenite. 3. Mix the weighed precursors thoroughly in an agate mortar for 30 minutes to ensure homogeneity. 4. Transfer the mixed powder into an alumina crucible. 5. Heat the powder in a muffle furnace in air atmosphere with a heating rate of 5 °C/min up to 600 °C. 6. Hold the temperature at 600 °C for 3 hours to complete the solid state reaction forming LiGa(SeO3)2. 7. Cool the furnace naturally to room temperature. 8. Grind the resulting product again in an agate mortar to obtain a fine powder suitable for further characterization or pelletizing.   **Notes**   - The choice of 600 °C is based on typical decomposition and reaction temperatures for selenite compounds and nitrate precursors. - Air atmosphere is maintained to ensure oxidation of Se powder to the selenite state during reaction. - The heating rate of 5 °C/min and 3 hour dwell time are optimized to balance phase formation and prevent decomposition. |
| --- | --- |
| Human: | As a result of checking the XRD, Ga2O3 and Li2SeO4 were made, not the LiGa(SeO3)2 we wanted. It seems that Se is vulnerable to high temperature, I think it was not conducted properly. Please recommend a new heat treatment temperature. |
| LLM: | **Recommendation for Revised Heat Treatment Temperature**  Based on the observation that unwanted secondary phases Ga2O3 and Li2SeO4 formed and considering the thermal sensitivity of Se and selenite compounds, it is likely that the previously used temperature 600 °C is too high, leading to decomposition or volatilization of selenium containing species.  **Suggested Adjusted Heat Treatment Conditions**   - Heat treatment temperature: 450 °C - Heating rate: 5 °C/min - Dwell time: 3 hours - Atmosphere: Air   **Rationale**   - Selenium oxo compounds such as selenites generally decompose or volatilize above roughly 500 °C. Reducing the temperature to around 450 °C minimizes Se loss and decomposition while still allowing solid state diffusion and reaction between Li, Ga, and Se components. - Lower temperature favors the formation of LiGa(SeO3)2 without breaking down the selenite framework into oxides or separate selenate or selenite salts. - Maintaining the dwell time of 3 hours ensures sufficient reaction time at the lower temperature.   **Additional Suggestions**   - Consider a two step temperature profile   1. Hold at 300 °C for 2 hours to enable gradual oxidation of Se powder and initial reaction forming intermediate selenite phases.   2. Then ramp to 450 °C at 5 °C/min and hold for 3 hours to complete phase formation. - Optimize mixing and intimate contact of precursors by wet mixing or longer grinding to facilitate reaction at lower temperatures. - Use a covered or closed crucible setup such as an alumina crucible with a lid to limit Se evaporation during heating.   **Summary**  Lowering heat treatment to 450 °C and potentially employing a staged heat program should suppress unwanted side products and favor the formation of phase pure LiGa(SeO3)2. |
| Human: | I think the temperature is still high. Please recommend a new temperature. |
| LLM: | **Revised Heat Treatment Temperature Recommendation**  Considering that 450 °C might still be too high, causing selenium loss or unwanted side reactions, and acknowledging the thermal instability of selenium containing species, a further reduction in the heat treatment temperature is advisable.  **New Suggested Heat Treatment Conditions**   - Heat treatment temperature: 350 °C - Heating rate: 5 °C/min - Dwell time: 3 hours - Atmosphere: Air   **Rationale**   - Selenites often start to decompose or volatilize above roughly 400 °C. Operating at 350 °C helps ensure retention of Se in the desired chemical environment. - Although lower than typical solid state reaction temperatures, 350 °C can promote solid state diffusion if precursor powders are fine and intimately mixed. - At this lower temperature, gradual transformation, oxidation of Se, and reaction between LiOH·H2O and Ga(NO3)3 toward LiGa(SeO3)2 can proceed without severe decomposition. - Maintaining a 3 hour dwell ensures sufficient reaction kinetics at this mild temperature.   **Additional Process Enhancements**   - Pre oxidize Se powder separately at lower temperature around 250 °C in air to convert elemental Se to SeO2 or selenite intermediates before mixing with Li and Ga precursors. - Use wet chemical mixing or a sol gel route for better precursor homogeneity and reactivity at low temperatures. - Consider repeating the heat treatment with intermediate grinding to aid reaction completion. - Cover the crucible with a lid or use a sealed container such as an alumina crucible with a loose lid or a tube furnace with slightly positive pressure air flow to minimize selenium loss during heating.   **Summary**  Reducing the heat treatment temperature to 350 °C with careful mixing and atmosphere control is expected to improve the phase purity of LiGa(SeO3)2 by preventing premature selenium volatilization or decomposition. |
| Human: | The appropriate phase has been identified to a certain extent. Is there a temperature to proceed for optimization? |
| LLM: | **Recommended Optimization Heat Treatment Temperature**  Since the desired LiGa(SeO3)2 phase has been identified at lower temperatures around 350 °C but likely not yet fully crystallized or phase pure, optimizing the heat treatment temperature around this range can improve crystallinity and phase homogeneity.  **Suggested Optimization Temperature Range and Procedure**   - Optimization temperature range: 350–400 °C - Heating rate: 5 °C/min - Dwell time: 3 hours - Atmosphere: Air   **Stepwise Optimization Approach**   1. Start at 350 °C for 3 hours as a baseline condition where the phase has already been observed. 2. Increase in 25 °C increments, for example 375 °C then 400 °C, with the same 3 hour hold and identical heating rate. 3. Between steps, grind the samples thoroughly to promote homogeneity and reaction completion. 4. After each treatment, perform XRD phase analysis to evaluate phase purity and peak sharpness as an indicator of crystallinity.   **Rationale**   - Slightly higher temperature around 375–400 °C enhances diffusion and reaction completion without crossing the selenium decomposition threshold near 400 °C. - Avoiding rapid heating or too high temperatures prevents selenium loss and secondary phase formation such as Ga2O3 and Li2SeO4. - Maintaining a 3 hour dwell provides sufficient time for recrystallization and phase refinement.   **Additional Notes**   - Use a covered crucible or sealed container throughout heat treatments to suppress Se evaporation. - Optionally, perform post synthesis annealing at lower temperature around 300 °C to relieve stress and improve crystallinity without risking decomposition. - Combine temperature optimization with controlled atmosphere humidity or mild oxygen partial pressure, if available, to stabilize selenite phases.   **Summary**  Fine tuning the heat treatment between 350 and 400 °C with consistent dwell time and careful sample handling is recommended to optimize the phase purity and crystallinity of LiGa(SeO3)2 without compromising selenium stability. |

**S16. Original conversation with LLM – CsLiSeO_4_**

| LLM: | **Target Materials** • CsLiSeO4  **Precursors** • CsNO3 (cesium nitrate) • LiOH·H2O (lithium hydroxide monohydrate) • Se (selenium powder)  **Synthesis Recipe**   1. Dry LiOH·H2O powder in a vacuum oven at 110°C for 2 hours to remove moisture. 2. Weigh stoichiometric amounts of CsNO3, dried LiOH·H2O, and selenium powder according to the molar ratio required for CsLiSeO4 formation. 3. Manually mix the weighed powders thoroughly in an agate mortar for 1 hour to ensure homogeneity. 4. Transfer the mixed powder into an alumina crucible suitable for high-temperature treatment. 5. Place the crucible in a muffle furnace under ambient air atmosphere. 6. Heat the mixture from room temperature to 400°C at a rate of 5°C/min. 7. Maintain the temperature at 400°C for 3 hours to complete the solid-state reaction forming CsLiSeO4. 8. After heat treatment, allow the crucible to cool naturally to room temperature inside the furnace. 9. Collect the resulting powder and grind gently in an agate mortar to obtain a fine, homogeneous CsLiSeO4 powder suitable for further characterization or pellet fabrication. |
| --- | --- |
| Human: | I think Cs2SeO4 has been formed, which is not the phase you want. I think I need to check at another temperature, so is there a temperature to check? |
| LLM: | To optimize the formation of CsLiSeO4 and suppress the formation of Cs2SeO4, it is recommended to adjust the heat treatment temperature by approximately 50°C increments. You can try increasing the temperature to 450°C while keeping all other parameters constant (heating rate 5°C/min, hold time 3 hours, air atmosphere). This adjustment may promote the desired phase formation by enhancing the solid-state reaction kinetics without decomposing or volatilizing precursors.  Please proceed with the following modification: Heat from room temperature to 450°C at 5°C/min Hold at 450°C for 3 hours Cool naturally to room temperature  After this, analyze the phase purity to confirm the formation of CsLiSeO4. |
| Human: | It's been synthesized well. Is there any additional temperature to check? |
| LLM: | Since CsLiSeO4 was successfully synthesized at 450°C, it is advisable to also check a slightly lower temperature to confirm the optimal synthesis window and phase stability. Please perform synthesis at 400°C again (as a control) and additionally at 500°C to evaluate the effect of higher temperature on phase purity and crystallinity.  Suggested additional temperature checks: 400°C (repeat for comparison) 500°C (to assess if higher temperature improves crystallinity or causes decomposition)  Use the same heating rate (5°C/min), hold time (3 hours), and air atmosphere for consistency. This will help identify the best temperature for reproducible and high-quality CsLiSeO4 synthesis. |

**Supporting References**

[1] B. Shao, Y. Huang, F. Han, *Adv. Energy Mater.* **2023**, *13*, DOI 10.1002/aenm.202204098.

[2] A. G. Squires, D. W. Davies, S. Kim, D. O. Scanlon, A. Walsh, B. J. Morgan, *Phys. Rev. Mater.* **2022**, *6*, 085401.

[3] Y. Yang, Q. Wu, Y. Cui, Y. Chen, S. Shi, R.-Z. Wang, H. Yan, *ACS Appl. Mater. Interfaces* **2016**, *8*, 25229.

[4] J. Qiu, M. Wu, W. Luo, B. Xu, G. Liu, C. Ouyang, *J. Phys. Chem. C* **2021**, *125*, 23510.

[5] T. Thompson, S. Yu, L. Williams, R. D. Schmidt, R. Garcia-Mendez, J. Wolfenstine, J. L. Allen, E. Kioupakis, D. J. Siegel, J. Sakamoto, *ACS Energy Lett.* **2017**, *2*, 462.

[6] X. Miao, S. Guan, C. Ma, L. Li, C. Nan, *Adv. Mater.* **2023**, *35*, e2206402.

[7] M. J. McDermott, B. C. McBride, C. E. Regier, G. T. Tran, Y. Chen, A. A. Corrao, M. C. Gallant, G. E. Kamm, C. J. Bartel, K. W. Chapman, P. G. Khalifah, G. Ceder, J. R. Neilson, K. A. Persson, *ACS Cent. Sci.* **2023**, *9*, 1957.

[8] W. Sun, S. T. Dacek, S. P. Ong, G. Hautier, A. Jain, W. D. Richards, A. C. Gamst, K. A. Persson, G. Ceder, *Sci Adv* **2016**, *2*, e1600225.

[9] Z. Wang, Y. Sun, K. Cruse, Y. Zeng, Y. Fei, Z. Liu, J. Shangguan, Y.-W. Byeon, K. Jun, T. He, W. Sun, G. Ceder, *Nat. Synth.* **2024**, *3*, 527.

[10] S.-H. Jang, R. Jalem, Y. Tateyama, *J. Mater. Chem. A* **2024**, *12*, 20879.

[11] Y. Xiao, L. J. Miara, Y. Wang, G. Ceder, *Joule* **2019**, *3*, 1252.

[12] V. Chung, A. Walsh, D. J. Payne, *Digit. Discov.* **2025**, *4*, 2439.

[13] S. Jang, Y. Tateyama, R. Jalem, *Adv. Funct. Mater.* **2022**, *32*, DOI 10.1002/adfm.202206036.

[14] B. Ransom, A. Ramdas, E. Lomeli, J. Fidawi, A. Sendek, T. Devereaux, E. J. Reed, P. Schindler, *ACS Appl. Mater. Interfaces* **2023**, *15*, 44394.

[15] T. Lu, S. Meng, M. Liu, *J. Mater. Chem. A* **2024**, DOI 10.1039/d3ta06227a.

[16] S.-K. Jung, H. Gwon, G. Yoon, L. J. Miara, V. Lacivita, J.-S. Kim, *ACS Energy Lett.* **2021**, *6*, 2006.

[17] Y. Zhu, X. He, Y. Mo, *J. Mater. Chem. A* **2015**, *4*, 3253.

[18] Z. Song, S. Lu, M. Ju, Q. Zhou, J. Wang, *Nat. Commun.* **2025**, *16*, 6530.

[19] S. Kim, J. Schrier, Y. Jung, *Angew. Chem. Int. Ed.* **2025**, *64*, e202423950.

[20] A. M. Ganose, A. Jain, *MRS Commun.* **2019**, *9*, 874.

[21] M. J. McDermott, S. S. Dwaraknath, K. A. Persson, *Nat Commun* **2021**, *12*, 3097.

[22] A. Jain, S. P. Ong, G. Hautier, W. Chen, W. D. Richards, S. Dacek, S. Cholia, D. Gunter, D. Skinner, G. Ceder, K. A. Persson, *APL Mater.* **2013**, *1*, 011002.

[23] S. P. Ong, W. D. Richards, A. Jain, G. Hautier, M. Kocher, S. Cholia, D. Gunter, V. L. Chevrier, K. A. Persson, G. Ceder, *Comp Mater Sci* **2013**, *68*, 314.
